# Kynurenine in IDO1^high^ cancer cell-derived extracellular vesicles promotes angiogenesis by inducing endothelial mitophagy in ovarian cancer

**DOI:** 10.1186/s12967-024-05054-5

**Published:** 2024-03-11

**Authors:** Xiang Ying, Xiaocui Zheng, Xiaoqian Zhang, Yujia Yin, Xipeng Wang

**Affiliations:** Department of Obstetrics and Gynecology, Xinhua Hospital, Shanghai Jiao Tong University School of Medicine, 1665 Kongjiang Rd, Yangpu District, Shanghai, 200092 China

**Keywords:** IDO1, Extracellular vesicles, Mitophagy, Angiogenesis, Ovarian cancer

## Abstract

**Background:**

Mitophagy, a prominent cellular homeostasis process, has been implicated in modulating endothelial cell function. Emerging evidence suggests that extracellular vesicles (EVs) participate in intercellular communication, which could modulate tumor angiogenesis, a hallmark of ovarian cancer (OC) progression. However, the underlying mechanisms through how EVs regulate endothelial mitophagy associated with tumor angiogenesis during OC development remain obscure.

**Methods:**

The effect of cancer cell-derived EVs on endothelial mitophagy and its correlation with tumor angiogenesis and OC development were explored by in vitro and in vivo experiments. Multi-omics integration analysis was employed to identify potential regulatory mechanisms of cancer cell-derived EVs on endothelial mitophagy, which is involved in tumor angiogenesis associated with OC development. These insights were then further corroborated through additional experiments. An orthotopic OC mouse model was constructed to assess the antiangiogenic and therapeutic potential of the Indoleamine 2,3 dioxygenase-1 (IDO1) inhibitor.

**Results:**

Cancer cell-derived EVs promoted tumor angiogenesis via the activation of endothelial mitophagy, contributing to the growth and metastasis of OC. The aberrantly high expression of IDO1 mediated abnormal tryptophan metabolism in cancer cells and promoted the secretion of l-kynurenine (L-kyn)-enriched EVs, with associated high levels of L-kyn in EVs isolated from both the tumor tissues and patient plasma in OC. EVs derived from IDO1^high^ ovarian cancer cells elevated nicotinamide adenine dinucleotide (NAD +) levels in endothelial cells via delivering L-kyn. Besides, IDO1^high^ ovarian cancer cell-derived EVs upregulated sirt3 expression in endothelial cells by increasing acetylation modification. These findings are crucial for promoting endothelial mitophagy correlated with tumor angiogenesis. Notably, both endothelial mitophagy and tumor angiogenesis could be suppressed by the IDO1 inhibitor in the orthotopic OC mouse model.

**Conclusions:**

Together, our findings unveil a mechanism of mitophagy in OC angiogenesis and indicate the clinical relevance of EV enriched L-kyn as a potential biomarker for tumorigenesis and progression. Additionally, IDO1 inhibitors might become an alternative option for OC adjuvant therapy.

**Graphical Abstract:**

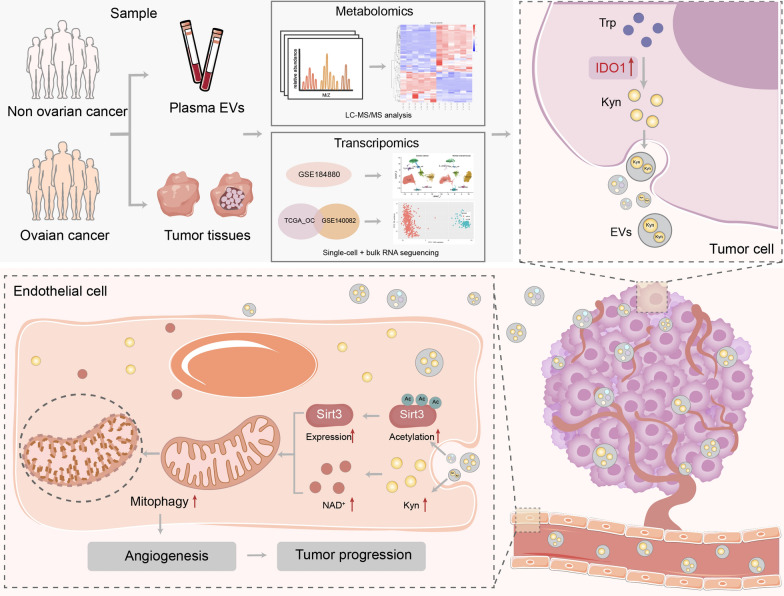

**Supplementary Information:**

The online version contains supplementary material available at 10.1186/s12967-024-05054-5.

## Introduction

Ovarian cancer (OC) is the tenth leading common cancer in females in the United States and is the most common cause of cancer-related death among female reproductive malignancies [1].Globally, approximately 314,000 new cases of OC were diagnosed, and almost 207,000 patients died from OC in 2020 according to the latest statistics [2].In China, the incidence and mortality rates of OC rank in the top ten among all female malignancies, with mortality rates increasing annually [2].OC often progresses rapidly, and the majority of OC patients are diagnosed at an advanced stage [3], characterized by widespread abdominal metastasis and malignant peritoneal ascites [4]. This late diagnosis, attributed to a lack of specific symptoms and effective biomarkers, significantly contributes to a poor prognosis. Despite advancements in the current treatment strategies, the 5-year survival rate of OC remains low [5, 6].Thus, it is urgent to explore the molecular pathogenesis of OC, which may provide novel targets in disease diagnosis and therapy.

Angiogenesis, which is essential for tumor growth and metastasis by supplying energy, is generally accepted to correlate with a poor prognosis in OC [7, 8]. For instance, previous studies revealed that extracellular vesicles (EVs) released by cancer cells could promote angiogenesis, resulting in tumor progression of OC [9–11]. Aberrant molecular expression of cancer cells also regulates angiogenesis [7, 8, 12], which affects tumor growth, metastasis, and treatment responses in OC. Accordingly, disrupting blood supply of tumors is considered an attractive potential therapeutic strategy. In recent years, the emergence and clinical application of novel antiangiogenic drugs and clinical application of antiangiogenic drugs have improved prognosis of OC patients [13, 14]. However, the responses to angiogenesis inhibitors displayed diverse in OC patients, partly owing to an uncompleted comprehension of tumor angiogenesis mechanisms. Thus, exploring the underlying molecular mechanisms of tumor angiogenesis may guide the development of novel diagnosis and treatment strategies for OC.

Mitochondria, known as the cellular powerhouses, play essential roles in energy metabolism and regulate various cellular functions and processes, including angiogenesis [15–17]. Mitophagy, a subtype of selective autophagy, is an evolutionarily preserved approach for maintaining cellular functions [18]. Emerging studies indicate that mitophagy correlates with tumorigenesis and progression by eliminating excessive or dysfunctional mitochondria, which is related to resistance and unfavorable prognoses [19–22]. Additionally, mitophagy also impacts endothelial cell function [23, 24]. For example, mitophagy could regulate endothelial senescence by upregulating FUNDC1 [25].Vascular injury could also be attenuated by PINK1/TAX1BP1-mediated mitophagy in endothelial cells [26]. However, to our knowledge, research exploring the role of mitophagy in modulating endothelial function in the context of tumor angiogenesis remains scarce.

As a crucial organelle for controlling cellular energy metabolism, mitochondrial homeostasis is susceptible to alterations caused by intracellular metabolic processes and extracellular metabolite levels. Mitophagy, one of the major mitochondrial quality control mechanisms, can be modulated by metabolites [27, 28]. Metabolic reprogramming of tumor cells could result in metabolic disturbances and abnormal metabolite accumulation [29, 30]. Such changes not only affect the transformed tumor cells but also impact stromal cells within the tumor microenvironment (TME), as well as the homeostasis of the organs, further propelling tumor progression [31–33]. There is a growing realization that metabolites play roles in processes like angiogenesis and immunity, thereby contributing to tumor progression [34, 35]. EVs, nanoparticles secreted by a variety of cell types, encompass heterogeneous multi-biological active substances, including RNAs, lipids, and etc. [36–39]. Recognized as pivotal messengers in intercellular communication, there is a growing appreciation that EVs can also transport metabolites [40], which may serve as biomarkers discovery and potential treatment targets in OC. However, studies on the regulation of EV metabolites in the TME and their functions in tumor angiogenesis are still in infancy.

Here, we hypothesized that endothelial mitophagy may participate in tumor angiogenesis and progression, which could be partly regulated by EV metabolites released from ovarian cancer cells. In this regard, we initially explored the effect of cancer cell-derived EVs on endothelial mitophagy associated with tumor angiogenesis and the progression of OC. By incorporating multi-omics analyses, we identified that abnormally upregulated Indoleamine 2,3 dioxygenase-1(IDO1) expression in cancer cells altered tryptophan metabolism, facilitating the release of l-kynurenine (L-kyn)-enriched EVs acquired from the tumor tissues and plasma in OC, which were further validated by larger clinical samples and extra experiments. Subsequently, we found that IDO1^high^ cancer cell-derived EVs indeed promoted endothelial mitophagy, which was linked to tumor angiogenesis and OC development. Mechanistically, IDO1^high^ cancer cell-derived EVs raised nicotinamide adenine dinucleotide (NAD +) levels in endothelial cells by transferring L-kyn and also upregulated sirt3 expression in endothelial cells through regulating acetylation modification. Moreover, an IDO1 inhibitor restrained endothelial mitophagy, impeding tumor angiogenesis and progression in the orthotopic OC mouse model. Altogether, our findings reveal the function and regulatory mechanism of mitophagy in tumor angiogenesis and would provide evidence to develop novel liquid biopsy biomarkers and therapeutic strategies.

## Materials and methods

### Patient samples

Human tissue microarrays (TMAs), comprising 80 benign ovarian cyst tissues and 115 OC tissues, were collected from benign ovarian cysts or OC patients between 2008 and 2018.Peripheral blood from OC and non-malignant patients was collected between 2008 and 2023.Ethics approval was obtained from Xinhua Hospital’s Ethics Committee.

### Cell culture

Human normal ovarian epithelial cell line (IOSE80), human ovarian cancer cell lines (A2780, SKOV3), human umbilical vein endothelial cells (HUVECs), and murine ovarian cancer cell line (ID8) were used in this study. IOSE80, A2780, SKOV3, and ID8 were cultured in DMEM (Gibco, USA) supplemented with 10% fetal bovine serum (cyagen, China) and 1% antibiotics (Thermo Fisher Scientific, USA). HUVECs were cultured in EBM2 complete medium (Lonza, Switzerland). All cells were maintained in a cell incubator at 37 ℃ with 5% CO_2_. The cell lines were authenticated by short tandem repeat (STR) profiling.

### EV isolation

To isolate EVs from cultured cell lines, the cells were initially rinsed with phosphate-buffered saline (PBS) (Servicebio, #G4202, China) prior to incubation in culture media devoid of EVs. After 3–4 days, the cultured supernatant was collected for further extracting EVs. To separate plasma, whole blood collected in EDTA was centrifugated at 1500*g* for 10 min at 4 ℃. Tissue explant culture followed a previously described method [41].

The conditioned medium of either cultured cells, tissue explants, or plasma underwent a series of centrifugations: first at 500*g* for 10 min, then 3000*g* for 20 min, and finally 12,000*g* for 20 min, all at 4 ℃. EVs from the above sources were then obtained by ultracentrifugation at 100,000*g* for 70 min at 4 ℃, followed by a wash in PBS (Servicebio, #G4202, China) and re-ultracentrifugation. The concentration of EVs was determined using BCA protein assay (Thermo Fisher Scientific, #23225, USA).

### EV identification

The morphology of EVs was visualized using transmission electron microscopy (TEM). Specifically, 10 μl EVs suspended in PBS were placed on copper grids for a duration of 1 min. These grids were then negatively stained with 10 μl of uranyl acetate for another minute. Following a brief period of air drying at room temperature (RT), the grids were visualized under a TEM (Hitachi, HT-7700, Japan) set at 100 kV. Additionally, the size distribution of EVs was assessed using NanoFCM (N30E, NanoFCM Inc., China). Western blot was performed to detect EV-associated markers CD9, CD63 and CD81. Details for the primary antibodies are available in the Additional file 1: Table S1.

### EV labeling and uptake

The isolated EVs were labeled with PKH26 (Sigma-Aldrich, #MIDI26, USA) according to the provided instructions. Briefly, the extracted EVs were mixed with 4μL PKH26 and incubated for a duration of 5 min. Subsequently, an equivalent volume of 1% bovine serum albumin (BSA) was added to quench the reaction. The labeled EVs were then separated via ultracentrifuging at 100,000*g* for 70 min, followed by a wash in PBS and another round of ultracentrifugation. Ultimately, the labeled EVs were co-cultured with endothelial cells for 12 h. Post-incubation, the endothelial cells were washed, fixed and visualized using fluorescence microscopy (Olympus, CKX41, Japan).

### Immunofluorescence (IF)

HUVECs (5 × 10^4^cells/cm^2^) were cultured in a confocal dish for 24 h and then treated with various interventions. Subsequently, the treated HUVECs were stained with MitoTracker Red (Cell Signaling Technology, # 8778S, USA) for 30 min at 37 ℃ and fixed with cold methanol for 15 min at – 20 ℃. After undergoing 3 washes with PBS, they were permeabilized in Triton X-100 (Sigma-Aldrich, # T8787, USA) and blocked with 1%BSA, 10% goat-serum, and 0.3% glycine for 1 h at RT. Then, the cells were incubated with the primary antibody against LC3 (1:200) (Cell Signaling Technology, #4108S, USA) at 4 ℃ overnight. Subsequently, they were incubated with the related secondary antibody. Confocal images were captured with Leica SP5 confocal microscope (Leica, Germany) and analyzed by Fiji ImageJ software.

### Western blotting and co-immunoprecipitation (CoIP) assay

Protein lysates extracted from EVs, cells or mitochondria were electrophoretically separated using the 10% SDS-PAGE and subsequently transferred onto polyvinylidene difluoride (PVDF) membranes. The membranes were then incubated with 5% BSA for 2 h at RT. Thereafter, they were exposed to primary antibodies listed in Additional file 1: Table S1 overnight at 4 ℃. Following this, the membranes underwent incubation for 1 h with secondary HRP-conjugated antibodies at RT. The targeted signals were visualized using a ECL substrate (Pierce, #32209, USA).

For the CoIP assay, the treated HUVECs were collected and lysed in IP lysis buffer (Pierce, #87788, USA). Cell extracts were incubated with anti-sirt3 (Abcam, #ab246522, USA) and Pierce Classic Magnetic IP/Co-IP Beads (Pierce, #88804, USA). The immunoprecipitate was separated from the supernatant using a magnetic frame for magnetic beads and subsequently washed three times in PBS containing 0.05% NP-40. The proteins eluted from the beads were boiled in 1 lysed SDS gel-loading buffer and resolved by 10% SDS-PAGE. Primary antibody details were described in the Additional file 1: Table S1.

### Mitochondria isolation

In order to isolate the mitochondria from HUVECs, the procedure outlined in the manufacture’s protocol for the mitochondrial isolation kit (Beyotime, #C3601, China) was followed.

### Quantitative real-time PCR (qRT-PCR)

Cells were treated with TRIZOL (Invitrogen, #15596018CN, USA) to isolate total RNA, which was then converted into cDNA using PrimeScript RT-PCR kit (Takara, #RR037A, Japan) and subjected to real-time PCR. The primers for mRNAs are listed in Additional file 1: Table S2. 2^−ΔΔCq^ method were performed to analyze the gene expression.

### Tube formation assay

HUVECs (5 × 10^4^/well) were seeded onto materigel (Corning, #356230, USA) coated 48-well plates. Then, EVs (10 μg/ml) obtained from different sources were added to the plates seeded with HUVECs. HCQ (100 μM) or PBS was added to the cell culture with EV-depleted FBS. After incubation for 6 h at 37 ℃ in a humidified atmosphere with 5% CO2, the tube structure formation was imaged using microscope. The Image J was used to quantify the tube length.

### Wound healing assay

HUVECs (5 × 10^5^/well) were seeded in the plates and grown to nearly complete confluency. Subsequently, wounds were induced with a 200 μl pipette tip. After being washed, the detached cells were subjected to EV-depleted FBS medium containing various experimental groups. Scratch images were observed by microscope at 24 h.

### Multicolor immunohistochemistry

Murine primary tumor tissues were initially fixed with 4% paraformaldehyde, followed by deparaffinization and antigen retrieval. Next, the segments were blocked with 3% BSA for 30 min at RT. Then, the specimens were incubated with primary antibodies anti-CD31(1:4000) (abcam, #ab281583, USA), translocase of outer mitochondrial membrane 20(Tomm20) (1:2000) (Proteintech, #66777-1-2g, China) and LC3(1:400) (abcam, #ab48394, USA) overnight at 4 ℃. Subsequently, they were exposed to the appropriate secondary antibodies. Following a thorough wash and staining with DAPI (Life Technologies, #D1306, USA), the sections were observed using an inverted fluorescence microscope (Nikon Eclipse CI-S, Japan).

### Immunohistochemistry (IHC)

Paraffin sections from TMA including 80 benign ovarian cyst tissues and 115 OC tissues, and murine ovarian cancer tissues were deparaffinized with xylene, anhydrous ethanol and alcohol. Subsequently, they were washed with distilled water. Following that, the sections underwent antigen retrieval using the EDTA antigen retrieval buffer (Servicebio, #G1203, China). Afterwards, endogenous peroxidase activity of the sections was inhibited with 3% hydrogen peroxide solution. Subsequently, the sections were blocked with 3% BSA. Then, corresponding primary antibodies (refer to Additional file 1: Table S1) were applied and incubated overnight at 4 ℃.Thereafter, the related secondary antibodies were incubated, followed by applying a DAB substrate kit (DAKO, #K5007, Denmark).Then, the nuclei were counter stained. After that, the sections were dehydrated and mounted, and observed through a microscope (CIC, XSP-C204, China).

### Hematoxylin–eosin (HE) staining

HE staining was performed to confirm murine OC major metastasis lesion including peritoneum and omentum following the manufacturer’s directions. A microscope was used to capture the images.

### Plasmids construction and cell transfection

For gene-overexpression, the IDO1 cDNA was cloned into pShuttle-CMV (Qbiogene, USA). For gene-knockdown, the short heparin sequence was cloned into reconstituted pShutle-U617 (Qbiogene, USA) and homologously recombined in bacteria BJ5183. The recombinant plasmids were propagated separately in A2780 cells to get IDO1-OE and IDO1-sh.

### Cellular ATP detection

Intracellular adenosine 5′-triphosphate (ATP) levels of the treated HUVECs were measured by an ATP assay kit (Beyotime, #S0027, China). Briefly, the ATP level of cell lysate was measured by detecting the luminescence using a microplate reader (Synergy HT, BIOTEK). To mitigate potential inaccuracies arising from protein content, the protein concentration of the lysate was measured using BCA kit, and the concentration of ATP was converted into nmol/mg protein.

### Mitochondrial reactive oxygen species (mito-ROS), mitochondrial membrane potential, NAD + /NADH, and kyn measurement

Mito-ROS levels, mitochondrial membrane potential, and NAD + /Nicotinamide adenine dinucleotide (NADH) of the HUVECs were detected by a Mito-SOX Red dye (Invitrogen, #M36008, USA), Tetramethyl rhodamine ethyl ester (TMRE) (Abcam, #ab287864, USA) and NDA + /NADH assay kit (Abcam, #ab65348, USA) following the instructions. Besides, plasma, EVs, supernatant and the HUVECs were detected for kyn concentrations using the ELISA kit (MyBioSource, #MBS495082, USA) following the manufacturer’s instructions.

### Animal orthotopic OC model

All of the mouse experiments were conducted following animal care and use guidelines from Xinhua Hospital, Shanghai Jiaotong University School of Medicine. C57BL/6 female mice were purchased from Jihui Laboratory Animal Breeding Ltd in Shanghai, China. 6 weeks old mice were used for constructing an orthotopic OC model. Briefly, 5 × 10^5^ ID8 cells in 10 μl PBS were orthotopically injected into the left ovary.

To explore EVs education in vivo, 10 μg EVs isolated from IOSE80, A2780, control or IDO1-OE A2780 were delivered by intravenous injection once every 2 days for 4 weeks beginning with two weeks after OC orthotopic construction. After 6 weeks, the mice were euthanized, and primary tumor and metastasis tissues were collected for further analysis.

To explore the effect of the IDO1 inhibitor in vivo, mice in the experimental group drank water with 1-Methyl-DL-tryptophan (IDO1 inhibitor) (Sigma-Aldrich, #860646, USA) at 2 mg/ml since two weeks after OC orthotopic construction. After 8 weeks, the mice were euthanized and cancer samples were collected for subsequent study.

### Untargeted LC–MS/MS metabolomics

Metabolites of EVs acquired from OC and non-OC plasma samples were extracted as follows. The EVs were resuspended with 400 μl prechilled 80% methanol by well vortex. Then the samples were incubated on ice and whirled for 30 s. After the sonification for 6 min, they were centrifuged and the supernatant was freeze-dried and dissolved with 10% methanol. Finally, the solution was injected into the LC–MS/MS system analysis [42, 43]. Further, the samples were detected by UHPLC-MS/MS analysis.

### Metabolomic data processing and statistical analysis

The acquired raw data files through UHPLC-MS/MS were processed by Compound Discoverer 3.1 (CD3.1, ThermoFisher) software. Statistical analyses were performed using the statistical software R (R version R-3.4.3), Python (Python 2.7.6 version) and CentOS (CentOS release 6.6). The KEGG database was utilized to annotate the metabolites. Principal components analysis (PCA) and Partial least squares discriminant analysis (PLS-DA) were conducted by metaX. Univariate analysis (*t*-test) was utilized to calculate the statistical significance. Differential metabolites were identified as VIP > 1 and *P* < 0.05 and fold change ≥ 2 or FC ≤ 0.5. Volcano plots were utilized to filter metabolites of interest which based on log_2_(Fold Change) and -logio(*p*-value) of metabolites by ggplot2 package. For clustering heat maps, the intensity areas of differential metabolites were normalized by z-scores and visualized by Pheatmap package in R. Functions and metabolic pathways were identified by the KEGG database.

### Bioinformatics analysis of public database

Single-cell RNA-seq (ScRNA-seq) data from GSE184880 (59,324 cells) including 7 OC samples and 5 non-OC ovary samples was downloaded from Gene Expression Omnibus (GEO) database. After filtering out low-quality cells, we obtained a total of 47,401 cells for subsequent analysis. Firstly, t-distributed stochastic neighbor embedding (tSNE) and uniform manifold approximation and projection (UMAP) were performed to reduce dataset dimensionality with Seurat for visualization. Unsupervised analysis was used for first-run clustering identification and Seurat pipeline was used for a second time. For epithelial cell sub-clusting analysis, effect correction was batched by Harmony (v.1.0). Seurat FindAllMarkers function distinguished differential expressed genes of clusters. KEGG enrichment analysis is a practical resource for studying gene functions and associated high-level genome functional information. To better understand the mechanisms of mRNAs, ClusterProfiler package (version: 3.18.0) in R was employed to analyze KEGG enrichment pathway. The R software ggplot2 package was used to draw the boxplot; the R software pheatmap package was used to construct the heatmap. For the determination of differences in tryptophan metabolism, gene sets variation analysis (GSVA) package was performed.

The TCGA OC dataset containing 422 OC samples, and GETx containing 180 non-OC ovary samples were used for the identification and validation of hub genes involved in tryptophan metabolism in OC. Firstly, limma package with a false discovery rate (FDR) < 0.05 and log2(Fold Change) > 1 was carried out for filtering out DEGs between OC and non-OC ovary samples. Tryptophan metabolism-related genes were identified by searching Molecular Signature Database (MSigDB) with “KEGG TRYPTOPHAN METABOLISM”. Univariate Cox regression analysis with a survival package with *P* < 0.05 was subsequently performed to identify tryptophan metabolism prognosis-related genes.

Bulk RNA-seq data from GSE140082 containing 199 OC samples and from GETx containing 180 non-OC ovary samples were included to validate the difference in *IDO1* expression between OC and non-OC ovary tissues.

The Human Protein Atlas Database (HPA) (https://www.proteinatlas.org) was performed to validate the difference in IDO1 protein expression between OC and non-OC ovarian tissues.

R software (v3.6.3) was performed for statistical analyses. Pearson or Spearman correlation was carried out for correlation matrices. Wilcoxon test was used for the comparisons between the two groups and K–M curves using a Log-rank test were conducted for comparing survival differences. Statistically significant was set at *P* < 0.05.

### Statistical analysis

Data were analyzed with SPSS 20.0 software (SPSS Inc., Chicago, IL, USA) and were presented as mean ± SD. Student’s t-test, one-way ANOVA, two-way ANOVA, and U-test were used for determining. A *P* < 0.05 indicates the statistical significance. Fiji ImageJ software was performed for image processing.

## Results

### Cancer cell-derived EVs activated endothelial mitophagy involved in OC angiogenesis and progression in vivo

**Fig. 1:**
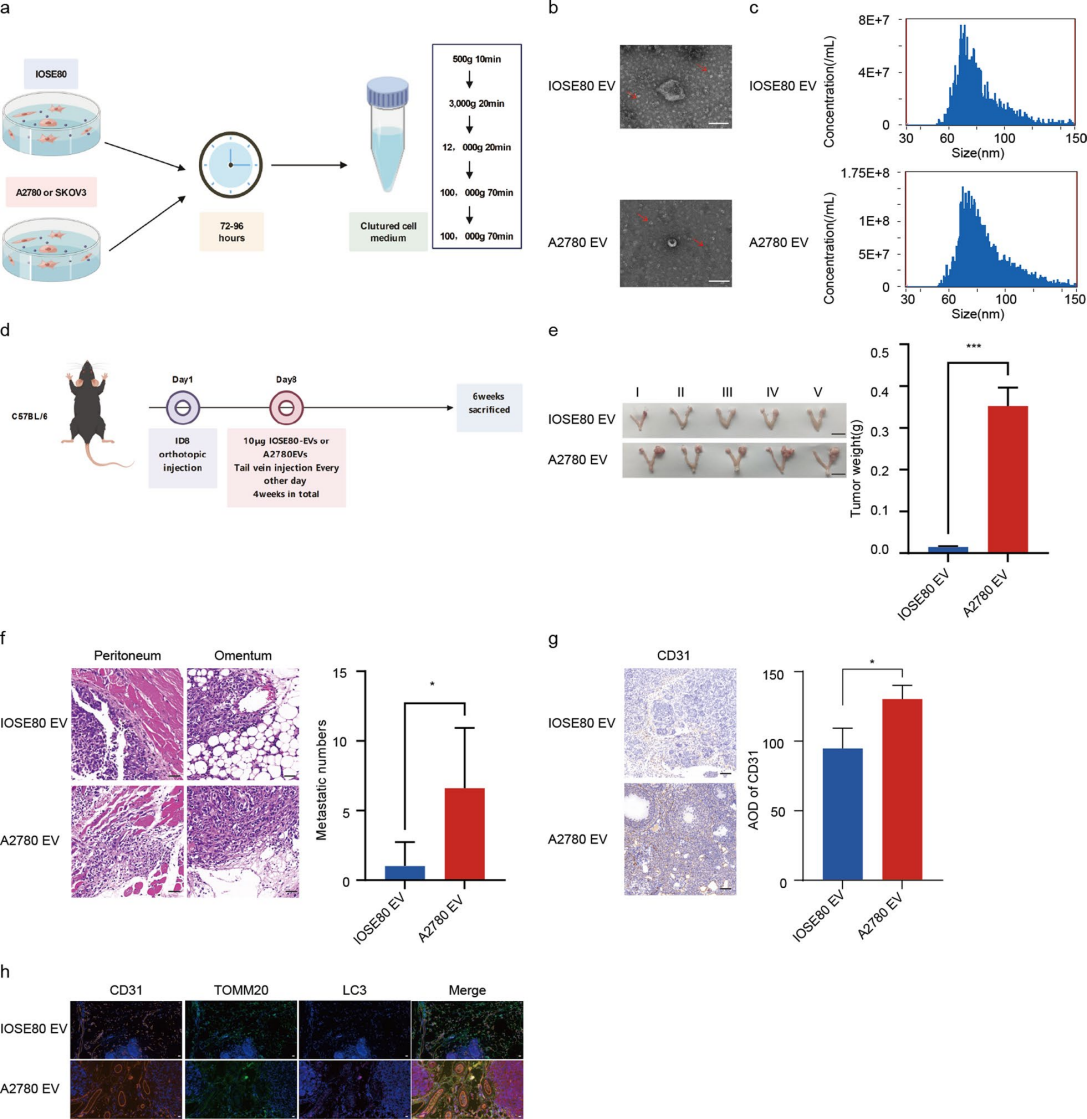
OC cell-derived EVs induced endothelial mitophagy related to tumor angiogenesis and progression in vivo. **a** Schematic illustration showing EVs isolation and purification process from the culture medium of normal ovarian epithelial cells (IOSE80) and ovarian cancer (OC) cells (A2780, SKOV3). **b** Representative images of EVs obtained from IOSE80 and A2780 cells by transmission electron microscope (TEM). Scale bar:100 nm. **c** Diameter ranges of EVs produced from IOSE80 and A2780 cells by NanoFCM. **d** Schematic diagram of orthotopic OC murine model educated with IOSE80-EVs or A2780-EVs (n = 5). **e** Representative images of the primary ovary tumor size in orthotopic OC model education with IOSE80-EVs or A2780-EVs. bar = 1 cm. (n = 5, Mean ± SD, t-test, ***P < 0.001). **f** Numbers of peritoneal metastases validated by HE staining in orthotopic OC model education with IOSE80-EVs or A2780-EVs. Scale bar:50 μm. (n = 5, Mean ± SD, t-test, *P < 0.05). **g** Immunohistochemistry (IHC) analysis of CD31 expression in primary tumor tissues of the orthotopic OC model education with IOSE80-EVs or A2780-EVs.Scale bar:50 μm. (n = 3, Mean ± SD, t-test, *P < 0.05). **h** Representative images of LC3, TOMM20and CD31 in primary tumor tissue sections of orthotopic OC model educated with IOSE80-EVs or A2780-EVs by immunofluorescence staining. Scale bar:20 μm

To explore the involvement of ovarian cancer cell-derived EVs in tumor angiogenesis and progression, we first isolated EVs from one human normal ovarian cell line and two ovarian cancer cell lines by ultracentrifugation (Fig. 1a). The characterization of the isolated EVs by transmission electron microscopy revealed the typical cup-shaped and rounded morphology (Fig. 1b). Diameters of the collected EVs by nanoparticle tracking analysis indicated that the average sizes between normal ovarian cell-derived EVs and ovarian cancer cell-derived EVs were similar (60–100 nm) (Fig. 1c).Besides, both cell supernatants-derived EVs expressed the classical EV markers, including CD9, CD81and CD63 (Additional file 1: Fig. S1a).

Then, we investigated whether ovarian cancer cell-derived EVs could impact tumor angiogenesis and progression in vivo. We implanted mouse ID8 OC cells orthotopically into the ovaries of C57BL/6 mice. Two weeks after the orthotopic ovarian tumor model construction, the mice were administered with 10 μg of normal ovarian cell-derived EVs or ovarian cancer cell-derived EVs intravenously once every two days for 4 weeks, after which the mice were euthanized (Fig. 1d). As expected, ovarian cancer cell-derived EVs significantly promoted the growth of primary tumors (Fig. 1e). Moreover, mice treated with ovarian cancer cell-derived EVs were presented with more metastatic tumor sites in the abdominal cavity, as confirmed by H&E staining (Fig. 1f). Further, the primary tumor tissues were detected by immunohistochemistry of CD31 staining (Fig. 1g), indicating ovarian cancer cell-derived EVs could obviously promote tumor angiogenesis in the mouse OC models. Emerging studies unravel that autophagy plays a vital role in regulating endothelial cells [44, 45], the building blocks of blood vasculature. Recent evidence also suggests that autophagy could modulate tumor angiogenesis [44, 46]. Mitophagy is a selective autophagy form for eliminating dysfunctional mitochondria specifically, involved in maintaining cellular homeostasis [47]. Accordingly, we speculated that mitophagy might be involved in the regulation of pro-angiogenic effects by ovarian cancer cell-derived EVs. Through multicolor immunohistochemistry, we found that ovarian cancer cell-derived EVs increased colocalization of CD31 with LC3 and TOMM20 in primary tumor tissues of the orthotopic OC mouse model (Fig. 1h). Together, the in vivo experiments disclosed that cancer-cell derived EVs activated endothelial mitophagy, thereby accelerating tumor angiogenesis and progression in OC.

### Cancer cell-derived EVs promoted angiogenesis by inducing endothelial mitophagy of OC in vitro

**Fig. 2:**
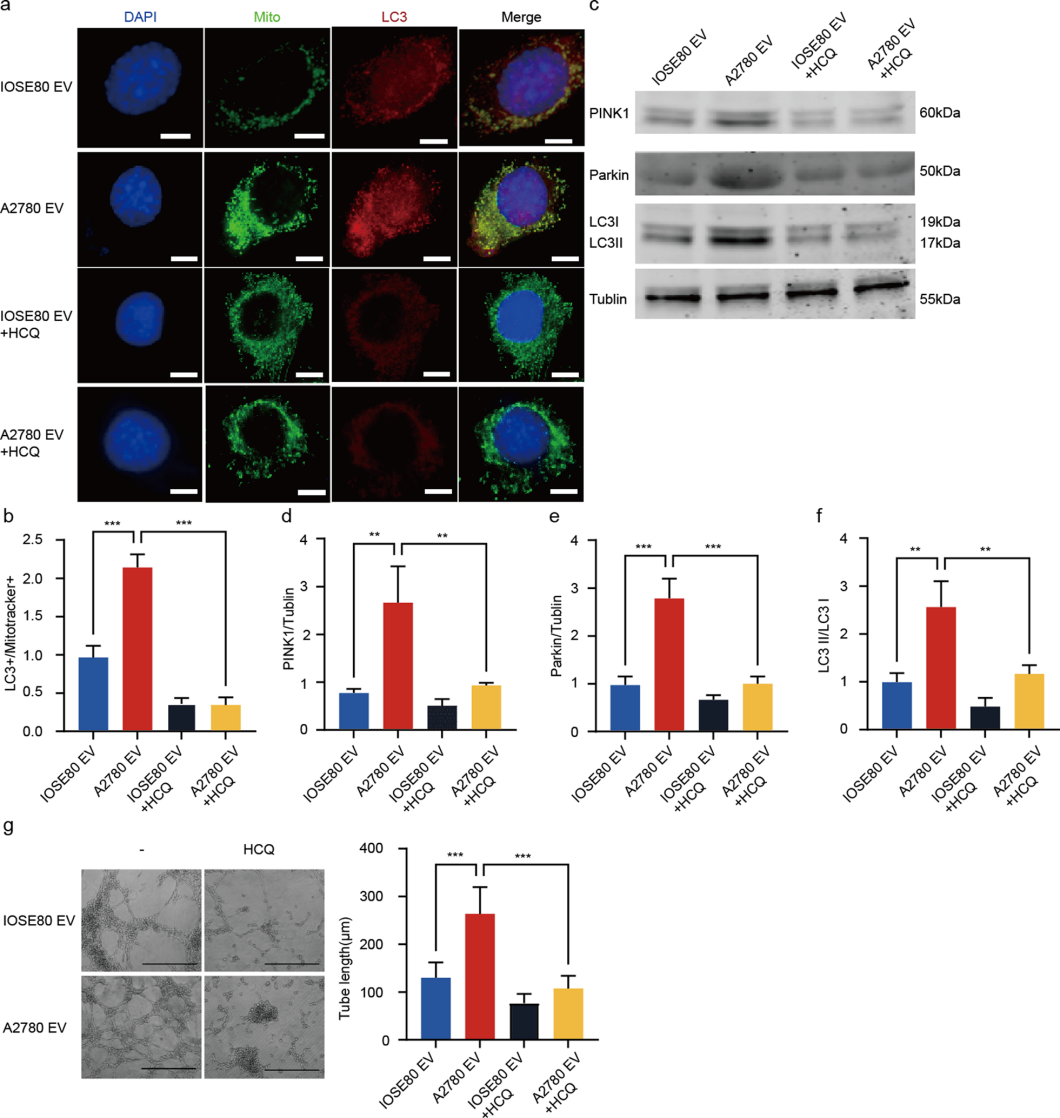
OC cell-derived EVs promoted angiogenesis by activating endothelial mitophagy in vitro. **a** Representative images of Mitotracker, LC3 and DAPI in endothelial cells incubated with different groups imaged by confocal microscopy. Scale bar: 10 μm. **b** LC3^+^/Mitotracker^+^ fluorescence intensity ratio in the different treated endothelial cells (n = 5, Mean ± SD, one-way ANOVA, ***P < 0.001). **c** Representative images of PINK1, Parkin, LC3 and Tublin protein expression in the different incubated endothelial cells by Western blot. **d**–**f** Quantification of PINK1, Parkin expression and LC3II/LC3I ratio in the different incubated endothelial cells by Western blot (n = 3, Mean ± SD, one-way ANOVA, ** P < 0.01,***P < 0.001). **g** Representative images and quantitative analysis of tube formation in different groups. Scale bar:500 μm. (n = 5, Mean ± SD, one-way ANOVA, ***P < 0.001)

To further clarify the role of mitophagy in the regulation of ovarian cancer cell-derived EVs in tumor angiogenesis, a co-culture system of EVs-endothelial cells was established in vitro. First, PKH26-labeled EVs were added to the medium of endothelial cells respectively, which were subsequently taken up by the endothelial cells (Additional file 1: Fig. S1b). Then, the formation of mitophagosomes (Mito Tracker + /LC3 + yellow puncta) observed by fluorescence confocal microscopy in endothelial cells were increased in the group treated with ovarian cancer cell-derived EVs, compared to those treated with normal ovarian cell-derived EVs. Furthermore, these phenomena can be reversed by a mitophagy inhibitor HCQ (Fig. 2a, b; Additional file 1: Fig. S2a). Besides, we also detected the protein levels of microtubule associated protein 1 light chain 3 alpha (LC3), PTEN induced kinase 1(PINK1) and parkin RBR E3 ubiquitin protein ligase (Parkin), classical markers of the mitophagy-associated pathway, in endothelial cells incubated with EVs. The results showed an increased LC3II/LC3I ratio, PINK1 and Parkin expression in the group treated with ovarian cancer cell-derived EVs when compared to that treated with normal ovarian cell-derived EVs. We discovered that the upregulation of the LC3II/LC3I ratio and the expression of PINK1 and Parkin could be inhibited by HCQ (Fig. 2c–f), further proving that ovarian cancer cell-derived EVs could trigger the mitophagy in endothelial cells. Additionally, the results of the tube formation assay showed that the tube length of endothelial cells treated with ovarian cancer cell-derived EVs was significantly longer than those treated with normal ovarian cell-derived EVs, suggesting that tube formation ability in HUVECs preconditioned by EVs from cancer cells was better than that of non-cancer cells. Moreover, HCQ could also rescue the tube formation-promoting effect of ovarian cancer cell-derived EVs in endothelial cells (Fig. 2g; Additional file 1: Fig. S2b). Altogether, these findings indicated that cancer cell-derived EVs raised pathological angiogenesis by triggering endothelial mitophagy in OC.

### Enrichment of L-kyn in EVs derived from OC patients and OC cells

**Fig. 3:**
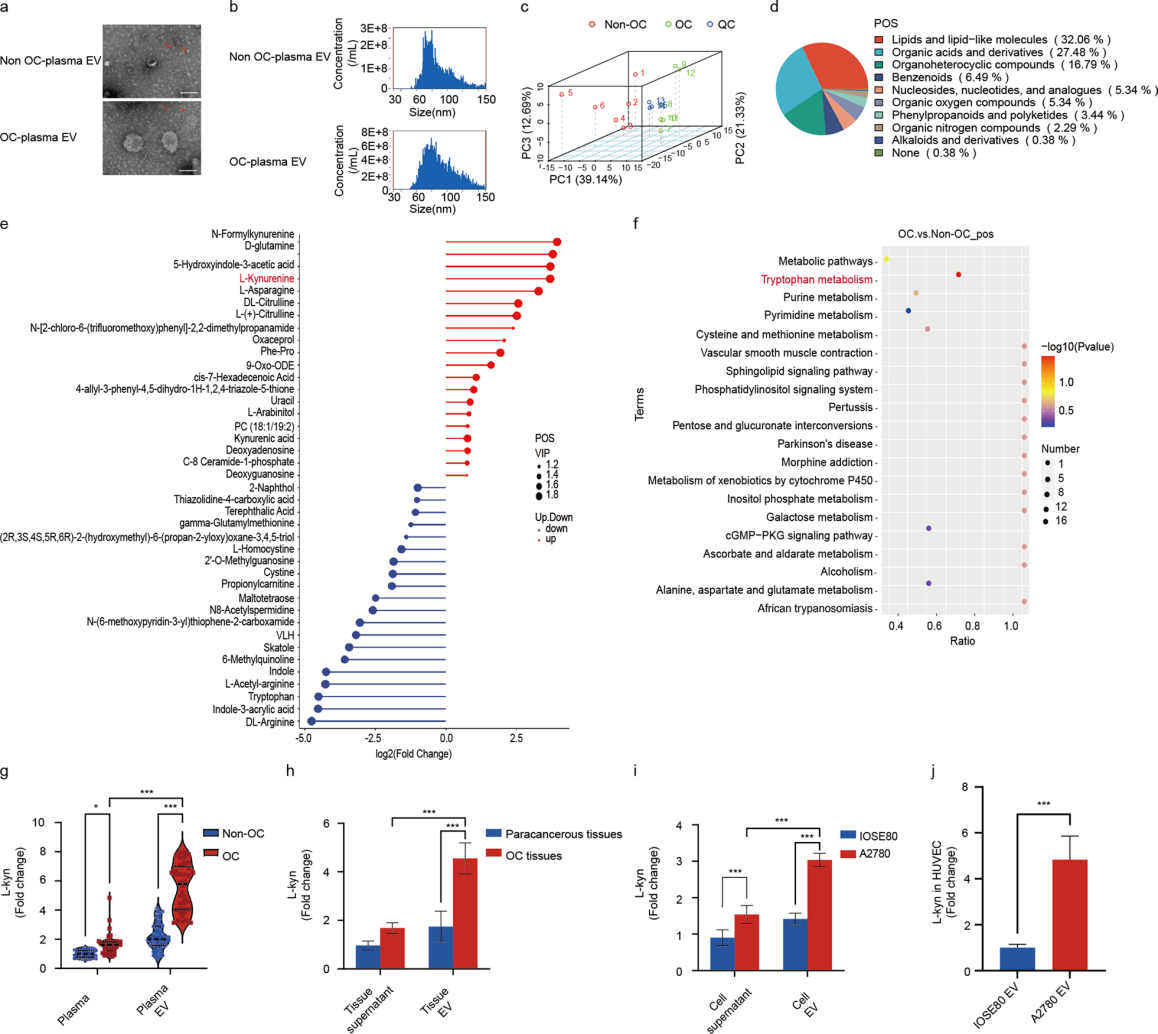
Enrichment of L-kyn in EVs acquired from OC patients and OC cells. **a** Representative images of EVs obtained from plasma with non-OC or OC patients by TEM. Scale bar: 100 nm. **b** Diameter ranges of EVs acquired from plasma with non-OC or OC patients by NanoFCM. **c** Principal component analysis (PCA) of total metabolite profiles with positive ion mode. **d** Pie chart depicting metabolite classification in positive ion mode. **e** Matchstick diagram showing top20 significant fold change of differential metabolites with positive ion mode between OC and non-OC plasma derived-EVs. **f** KEGG pathway enrichment analysis based on differentially accumulated metabolites with positive ion mode between OC and non-OC plasma derived-EVs. **g** Violin diagram showing level of L-kyn in plasma and plasma derived-EVs of non-OC and OC patients determined by ELISA (n = 40, two-way ANOVA, *P < 0.05,***P < 0.001). **h** Level of L-kyn in tissue supernatant and tissue derived-EVs of OC tissues and the corresponding para cancer tissues (n = 4, Mean ± SD, two-way ANOVA, ***P < 0.001). **i** Level of L-kyn in cell culture supernatant and cell derived-EVs of normal ovarian epithelial cells (IOSE80) and ovarian cancer cells (A2780) (n = 5, Mean ± SD, two-way ANOVA, ***P < 0.001). **j** Level of L-kyn in the endothelial cells treated with EVs obtained from normal ovarian epithelial cells (IOSE80) and ovarian cancer cells (A2780) (n = 5, Mean ± SD, t-test, ***P < 0.001). For **c**–**f**, all n = 6 plasma-derived EVs from patients with OC or non-OC were performed for analysis

The components and functions of EVs usually reflect the characteristics of their original cells [36, 48].Metabolic reprogramming is a hallmark that drives tumor development including ovarian cancer [49, 50].Emerging evidence implies that mitophagy could be modulated by metabolites [51, 52]. Here, we hypothesized that the mitophagy of endothelial cells, associated with pathological tumor angiogenesis, might be regulated by abnormal metabolites enriched in EVs released from OC. To screen the metabolite contents of EVs involved in ovarian cancer, plasma-derived EVs from OC and non-OC patients were isolated and validated (Fig. 3a, b; Additional file 1: Fig. S3a). Then, LC–MS/MS was performed on the plasma-derived EVs from OC and non-OC patients to explore the metabolomic features. Quality control (QC) of samples was initially conducted to ensure the data were satisfactory for downstream analysis (Additional file 1: Fig. S3b). The partial least squares discrimination analysis (PLS-DA) and principal component analysis (PCA) of all EV samples showed that the metabolic composition of plasma-derived EVs from OC and non-OC patients were extremely dissimilar (Fig. 3c; Additional file 1: Fig. S3c, d). The metabolomic results showed that lipids and lipid-like molecules and organic acids and derivatives were the main metabolic components in plasma-derived EVs (Fig. 3d; Additional file 1: Fig. S3e). Moreover, we compared the differences in the metabolites between plasma-derived EVs of OC and non-OC patients (Additional file 1: Fig. S3f). We noticed that L-kyn, a crucial intermediate product of tryptophan catabolism, was significantly increased in plasma-derived EVs from the OC group (Fig. 3e). Furthermore, Kyoto Encyclopedia of Genes and Genomes (KEGG) pathway analysis of both cationic and anionic metabolites also indicated that the metabolites highly enriched in plasma-derived EVs of OC patients were related to tryptophan metabolism (Fig. 3f; Additional file 1: Fig. S3g).Besides, we performed ELISA assays of L-kyn in EVs derived from plasma of OC and non-OC patients using another validation cohort and further confirmed that the level of L-kyn was significantly elevated in plasma-derived EVs from OC patients compared to those from non-OC. Interestingly, L-kyn was more enriched in the plasma-derived EVs than in the corresponding plasma (Fig. 3g). Thus, we suspected that there is an association between the enrichment of L-kyn in EVs and OC.

As reported, circulating EVs in patients diagnosed with solid tumors are often released from the cellular components in the TME or directly from the cancer cells [36, 53]. So, we then explored whether EVs enriched with L-kyn were released from cancer cells. Not surprisingly, L-kyn levels were consistently higher in OC tissue-derived EVs than that in the para-cancerous tissue-derived EVs (Fig. 3h), and L-kyn was also enriched in ovarian cancer cell-derived EVs compared with normal ovarian cell-derived EVs (Fig. 3i). Consistently, L-kyn was also enriched in the EVs acquired from different sources including tissues and cells, compared with the corresponding culture supernatant. Furthermore, we investigated the L-kyn level in the endothelial cells treated with EVs derived from the ovarian cancer cell line(A2780) and normal ovarian cell line (IOSE80), and ELISA assay showed an increased level of L-kyn in the endothelial cells with exposure to ovarian cancer cell-derived EVs (Fig. 3j). Taken together, these findings indicated that L-kyn, packaged in ovarian cancer cell-derived EVs, may be an important metabolite regulating tumor angiogenesis in OC.

### Aberrant tryptophan metabolism of tumor cells informed by OC scRNA-seq data

**Fig. 4:**
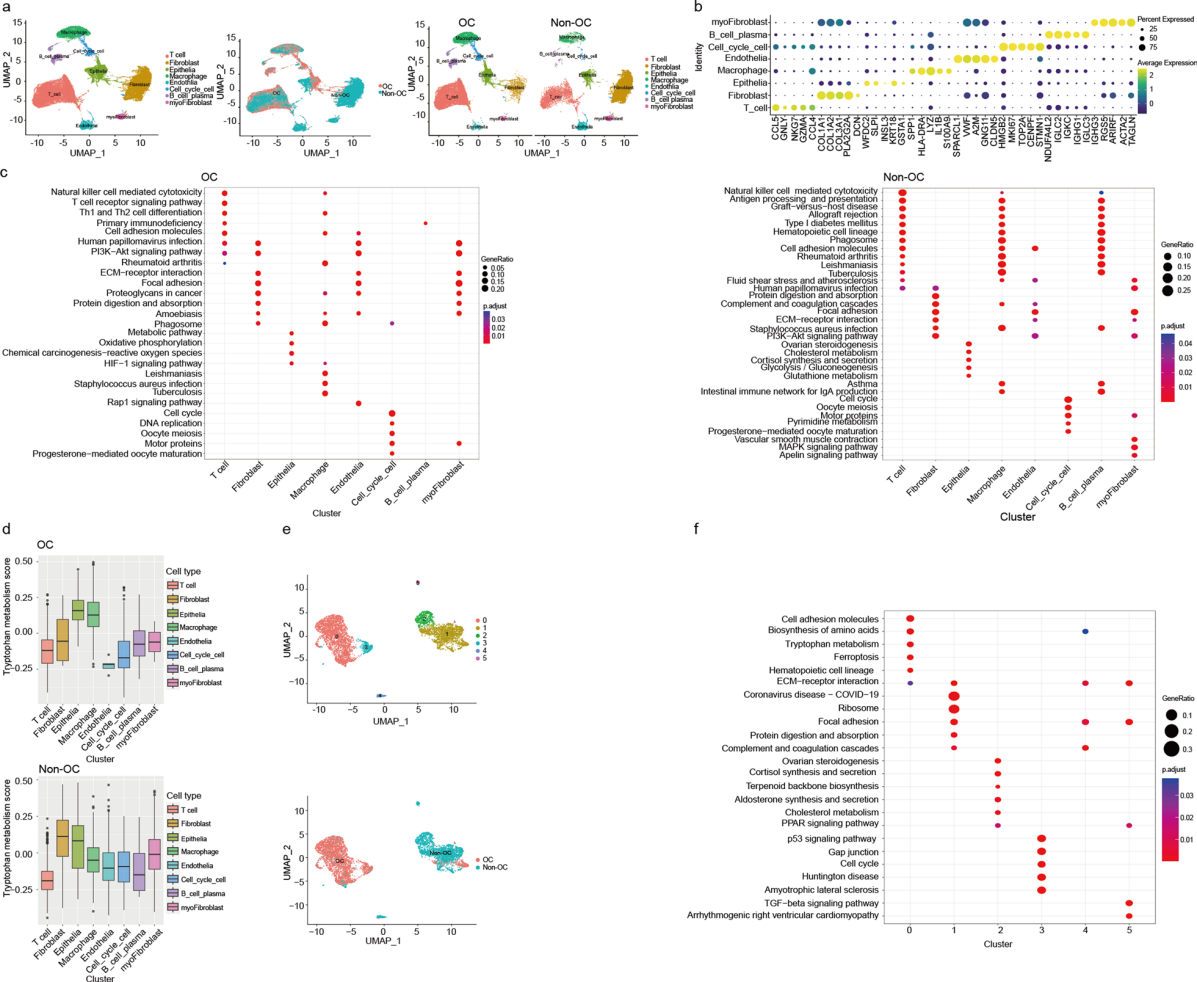
Tryptophan metabolic reprogramming of tumor cells discerned by OC scRNA-seq data. **a** Uniform Manifold Approximation and Projection (UMAP) plot revealing 8 clusters by integrating analysis. **b** Heat map showing marker genes of annotation in major cell clusters with OC and non-OC samples. **c** KEGG pathway enrichment analysis based on differentially expressed genes in major cell clusters of OC and non-OC ovary samples. **d** Gene set variation analysis (GSVA) revealing tryptophan metabolism-related genes enriched in major cell clusters of OC and non-OC samples. **e** UMAP plot showing subclusters of epithelial ovarian cancer cells and normal epithelial ovarian cells with OC and non-OC samples. **f** KEGG pathway enrichment analysis based on differentially expressed genes in subclusters of epithelial cells. For **a**–**f**, all n = 7 OC and n = 5 non-OC samples obtained from GSE184880 dataset were used for analysis

Previous findings indicated that EVs highly enriched with L-kyn were detected in the plasma of OC patients, as well as in primary ovarian cancer tissues, and ovarian cancer cell lines. L-kyn-enriched EVs might be released from ovarian cancer cells. To further identify whether tumor cells are major sources of L-kyn-enriched EVs, previous scRNA-seq data of OC were analysed [54]. After integrated analysis of scRNA-seq data including 7 primary ovarian tumor samples and 5 non-malignant ovarian samples, a total of 47,401 cells were divided and clustered into 8 major clusters according to classical markers, indicated by both tSNE and UMAP plots (Fig. 4a, b and Additional file 1: Fig. S4a). We noticed that both OC tissues and non-OC tissues harbored a certain fraction of epithelial cells (Additional file 1: Fig. S4b). Then, the characteristics of each cell type in primary ovarian cancer ecosystems were uncovered using KEGG. Gene enrichment of epithelial cells in OC was related to metabolic pathways, oxidative phosphorylation, chemical carcinogenesis-reactive oxygen species, HIF-1 signaling pathway while gene enrichment of epithelial cells in non-OC were associated with ovarian steroidogenesis, cholesterol metabolism, cortisol synthesis and secretion, glycolysis/gluconeogenesis and glutathione metabolism (Fig. 4c). Previous metabonomic profiles indicated that the differential highly enriched metabolites of plasma derived-EVs from OC patients compared to those from non-OC patients were mainly clustered into pathways of increased tryptophan metabolism. Therefore, we performed GSVA to explore the enrichment scores of tryptophan metabolism-related genes in eight different cell clusters between OC and non-OC. The results revealed that epithelial cells and macrophages were two major clusters presenting high levels of tryptophan metabolism in OC. Considering that malignant epithelial cells account for the largest proportion of cells in the OC tissues, we further compared the activity of tryptophan metabolism in normal epithelial cells and tumor cells. The result showed that ovarian cancer cells represented a higher level of tryptophan metabolism than normal epithelial cells (Fig. 4d). Then, features of epithelial cells were continued to be characterized and 6 different cell clusters were identified. Cluster 0 and cluster 3 were the main subclusters of epithelial cells in the primary ovarian tumor microenvironment (Fig. 4e; Additional file 1: Fig. S4c). Differentially expressed genes (DEGs) among the epithelial cell subclusters were analyzed and these genes were further carried out for functional annotation through KEGG pathway enrichment analysis (Fig. 4f). Notably, KEGG analysis showed that genes highly expressed in cluster 0 were enriched in the pathway of tryptophan metabolism. Together, these findings suggested that ovarian cancer cells may exhibit tryptophan metabolic disorders.

### Abnormal upregulation of *IDO1* expression related to tryptophan metabolic disorder in tumor cells of OC

**Fig. 5:**
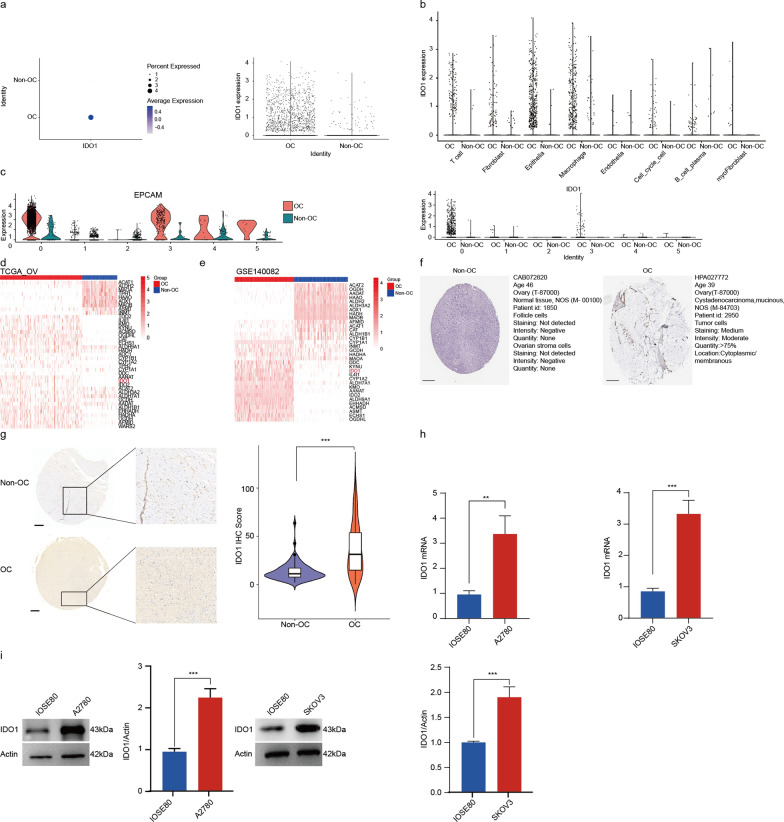
Aberrant *IDO1* upregulation involved in tryptophan metabolic reprogramming of OC cells. **a** Dot plot showing *IDO1* expression of OC and non-OC samples. **b** Visualization of *IDO1* expression in major cell clusters of OC and non-OC ovarian samples. **c** Visualization of *IDO1* expression in epithelial cell subclusters with OC and non-OC ovarian samples. **d** Heatmap showing *IDO1* expression in OC samples from OCGA_OV dataset and non-OC samples from GETx dataset. **e** Heatmap showing *IDO1* expression in OC samples from GSE140082 dataset and non-OC samples from GETx dataset. **f** Immunohistochemistry analysis of IDO1 protein expression between OC and non-OC ovarian tissues from the human protein atlas (HPA)database. Scale bar:200 μm. **g** Immunohistochemistry detecting of IDO1 protein expression in tissue microarray composed of OC (n = 115) and non-OC (n = 80) ovarian tissues from our hospital. Scale bar:200 μm. (u-test, ***P < 0.001). **h** *IDO1* mRNA expression between non-OC cells (IOSE80) and OC cells (A2780, SKOV3) was detected by qRT-PCR (n = 3, Mean ± SD, t-test, **P < 0.01, ***P < 0.001). **i** IDO1 protein expression between non-OC cells (IOSE80) and OC cells (A2780, SKOV3) was detected by Western blot (n = 3, Mean ± SD, t-test, ***P < 0.001). For **a**–**c**, all n = 7 OC and n = 5 non-OC samples obtained from GSE184880 dataset were used for analysis

IDO1 is a rate-limiting enzyme that plays an essential role in governing the tryptophan-kynurenine pathway [55]. Therefore, we further analyzed the expression level of *IDO1* in different clusters using previous OC scRNA-seq data. Not surprisingly, the *IDO1* expression level was higher in OC tissues than that in normal ovarian tissues (Fig. 5a). To explore which cell type contributed to the high level of *IDO1* in the TME, we investigated the *IDO1* expression among all eight distinct clusters. The result showed that the expression level of *IDO1* in epithelial cells and macrophages was significantly higher than in other clusters, consistent with previous findings that these two clusters exhibited elevated levels of genes associated with tryptophan metabolism (Fig. 5b). Additionally, the *IDO1* expression level in cluster 0 and cluster 3, mostly related to malignant epithelial cells, was significantly higher compared to other epithelial cells (Fig. 5c; Additional file 1: Fig. S4d).

Moreover, we compared the expression levels of *IDO1* between OC tissues and non-OC tissues using the TCGA ovarian cancer cohort, the previous RNA-seq dataset (GSE140082) and GETx. First, we screened out DEGs between OC tissues (from the TCGA ovarian cancer cohort) and non-OC ovarian tissues (from GETx) (Additional file 1: Fig. S5a). Among them, 39 genes including *IDO1* were closely related to tryptophan metabolism of OC in the TCGA ovarian cancer cohort (Fig. 5d), and 34 genes, including *IDO1*, were cohesively associated with tryptophan metabolism of OC in the GSE140082 cohort (Fig. 5e). Afterward, univariate Cox regression analysis was performed to evaluate the prognostic value of each tryptophan metabolism-related gene in the TCGA ovarian cancer cohort, with *IDO1, ALDH3A2, HADHA, OGDHL* associated with risk values, and CAT, WARS1 involved in protective factors (Additional file 1: Fig. S5b). Furthermore, we assessed IDO1 protein expression in OC tissues and non-OC tissues by the HPA database and the tissue microarray from our hospital through immunohistochemistry. Consistently, HPA database analysis and our tissue microarray showed that IDO1 protein levels in OC tissues were higher than those in benign ovary tissues, as indicated by IHC scores (Fig. 5f, g). These findings further confirmed the previous results indicated by scRNA-seq analysis. Additionally, we detected the mRNA and protein expression level of IDO1 in common ovarian cancer cell lines (A2780, SKOV3) and normal ovarian cell line using qRT-PCR and Western blot. As expected, IDO1 was highly upregulated in ovarian cancer cell lines (Fig. 5h, i). Above all, these evidences indicated that abnormally elevated *IDO1* expression of tumor cells could accelerate tryptophan metabolism in OC, which was related to the release of L-kyn enriched EVs.

### IDO1^high^ cancer cell-derived EVs activated endothelial mitophagy involved in OC angiogenesis and progression

**Fig. 6:**
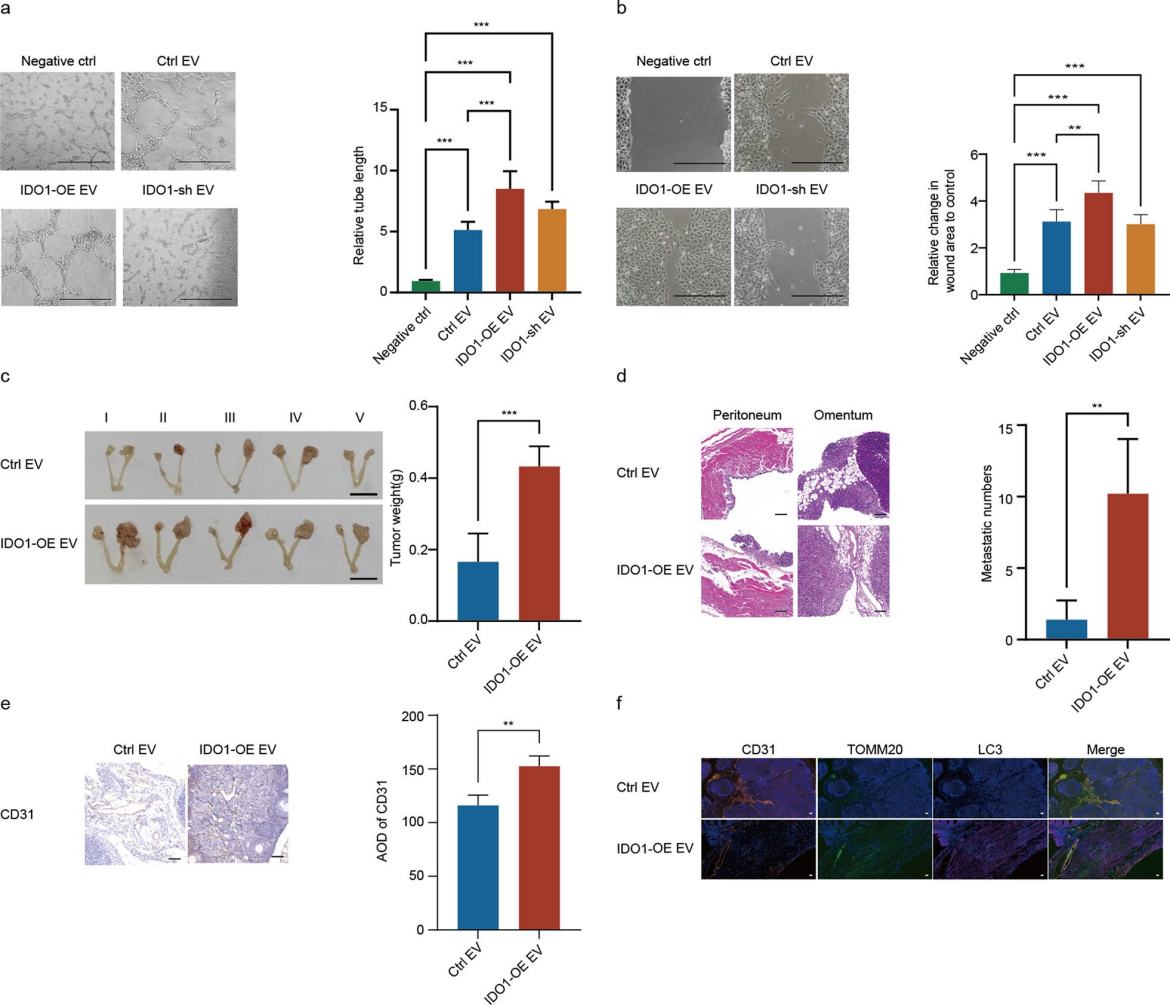
IDO1^high^ OC cell-derived EVs promoted tumor angiogenesis and development. **a** Representative images and quantitative analysis of tube formation assay for endothelial cells incubated with negative control (Negative ctrl), IDO1-control A2780 EVs (ctrl EV),IDO1-overexpression A2780 EVs(IDO1-OE EV) or IDO1-knockdown A2780 EVs(IDO1-sh EV).Scale bar:500 μm. (n = 5, Mean ± SD, one-way ANOVA, ***P < 0.001). **b** Representative images and quantitative analysis of wound healing assay for endothelial cells treated with Negative ctrl, ctrl EV, IDO1-OE EV or IDO1-sh EV. Scale bar:500 μm. (n = 5, Mean ± SD, one-way ANOVA, **P < 0.01, ***P < 0.001). **c** Representative images of the primary ovary tumor size in orthotopic OC model education with Ctrl EV or IDO1-OE EV. bar = 1 cm. (n = 5, Mean ± SD, t-test, ***P < 0.001). **d** Numbers of peritoneal metastases validated by HE staining in orthotopic OC model education with Ctrl EV or IDO1-OE EV. Scale bar:50 μm. (n = 5, Mean ± SD, t-test, **P < 0.01). **e** IHC analysis of CD31 expression in primary tumor tissues of the orthotopic OC model education with Ctrl EV or IDO1-OE EV. Scale bar:50 μm. (n = 3, Mean ± SD, t-test, **P < 0.01). **f** Representative images of LC3, TOMM20and CD31 in primary tumor tissue sections of orthotopic OC model educated with Ctrl EV or IDO1-OE EV by immunofluorescence staining. Scale bar:20 μm

Based on the above findings, we speculated that the abnormal expression of *IDO1* associated with tryptophan metabolic disorder of tumor cells, was involved in the secretion of L-kyn enriched EVs, which might regulate tumor angiogenesis and development through mitophagy. To verify this hypothesis, IDO1-overexpression and -knockdown ovarian cancer cell lines were constructed. Further, the overexpression and knockdown efficiency of IDO1 in ovarian cancer cell lines were verified by qRT-PCR and Western blot (Additional file 1: Fig. S6a, b). Then, endothelial cells were treated with EVs obtained from IDO1-overexpression and –knockdown ovarian cancer cells to perform tube formation and wound healing assay. The results showed that IDO1^high^ ovarian cancer cell-derived EVs induced endothelial migration and tube formation (Fig. 6a, b) in vitro.

To further explore the impact of EVs from IDO1^high^ ovarian cancer cells on tumor angiogenesis and development in vivo, we developed an orthotopic OC mouse model as previously described. As anticipated, IDO1^high^ ovarian cancer cell-derived EVs increased the weight and metastases of tumors (Fig. 6c, d), contributing to enhanced tumor angiogenesis (Fig. 6e). Besides, multicolor immunohistochemistry for primary tumor tissues unveiled enhanced endothelial mitophagy induced by IDO1^high^ ovarian cancer cell-derived EVs in the orthotopic ovarian cancer mouse model (Fig. 6f). Therefore, the above findings suggested that IDO1^high^ ovarian cancer cell-derived EVs induced endothelial mitophagy, which might correlate with tumor angiogenesis and progression.

### IDO1^high^cancer cell-derived EVs induce angiogenesis through activating endothelial mitophagy in OC

**Fig. 7:**
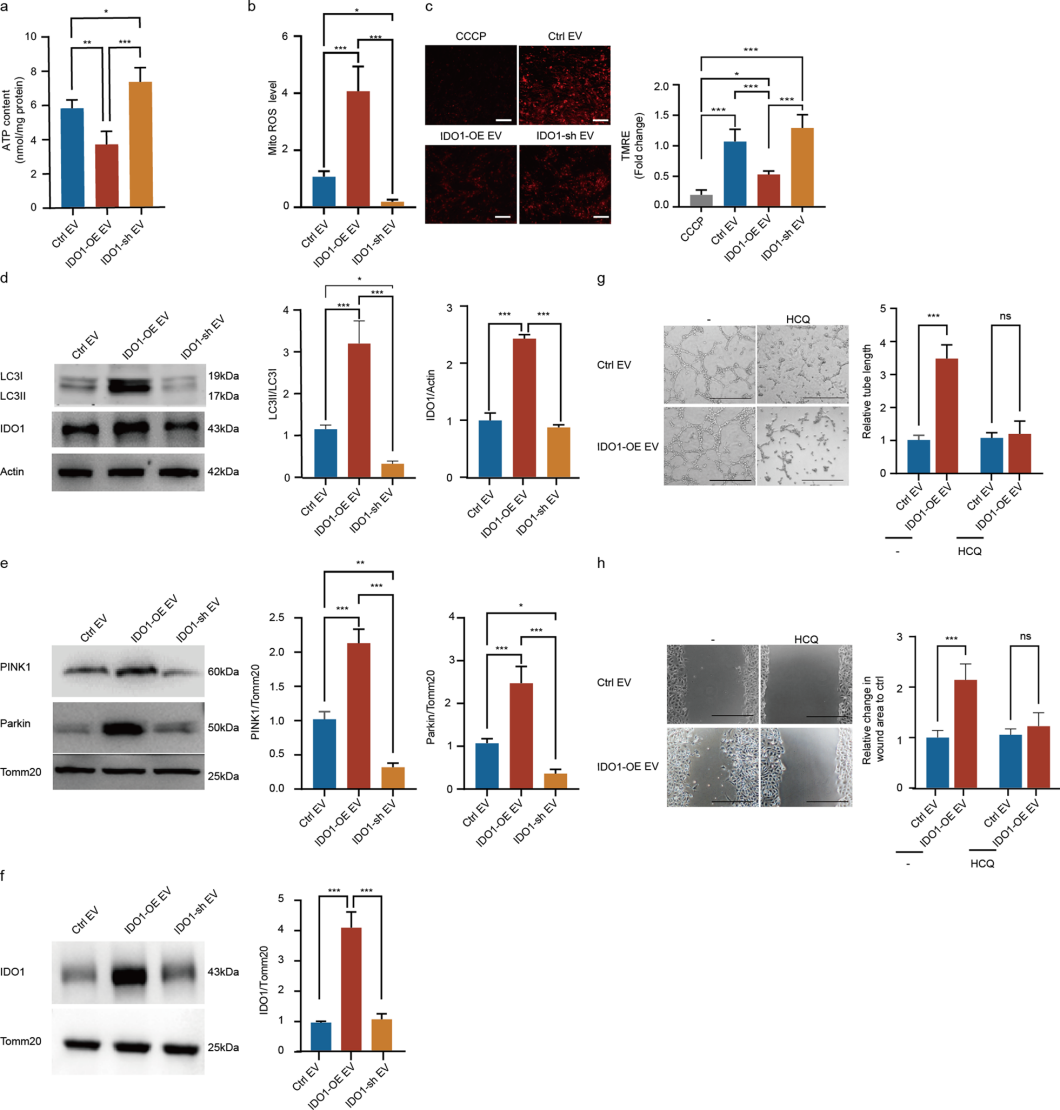
IDO1^high^ OC cell-derived EVs facilitated tumor angiogenesis by triggering PINK1-Parkin-mediated mitophagy of endothelial cells. **a** Intracellular ATP levels of the endothelial cells incubated with ctrl EV, IDO1-OE EV or IDO1-sh EV (n = 5, Mean ± SD, one-way ANOVA, *P < 0.05,**P < 0.01,***P < 0.001). **b** MitROS production of the endothelial cells incubated with ctrl EV, IDO1-OE EV or IDO1-sh EV (n = 5, Mean ± SD, one-way ANOVA, *P < 0.05,**P < 0.01,***P < 0.001). **c** Mitochondria membrane potential of the endothelial cells incubated with CCCP, ctrl EV, IDO1-OE EV or IDO1-sh EV. Scale bar:10 μm. (n = 5, Mean ± SD, one-way ANOVA, *P < 0.05, ***P < 0.001). **d** Representative images and quantification of LC3II/LC3I ration and IDO1 protein expression in the endothelial cells treated with ctrl EV, IDO1-OE EV or IDO1-sh EV by Western blot (n = 3, Mean ± SD, one-way ANOVA, *P < 0.05, ***P < 0.001). **e** Representative images and qualification of PINK1, Parkin protein expression in the mitochondria isolated from the endothelial cells treated with ctrl EV, IDO1-OE EV or IDO1-sh EV by Western blot (n = 3, Mean ± SD, one-way ANOVA, *P < 0.05, **P < 0.01, ***P < 0.001). **f** Representative images and qualification of IDO1 protein expression in the mitochondria isolated from the endothelial cells treated with ctrl EV, IDO1-OE EV or IDO1-sh EV by Western blot (n = 3, Mean ± SD, one-way ANOVA, ***P < 0.001). **g** Representative images and quantitative analysis of tube formation in the different treated endothelial cells. Scale bar:500 μm. (n = 5, Mean ± SD, one-way ANOVA, ***P < 0.001, *ns.* no significance). **h** Representative images and quantitative analysis of wound healing assay for the endothelial cells in different groups. Scale bar:500 μm. (n = 5, Mean ± SD, one-way ANOVA, ***P < 0.001, *ns.* no significance)

To further confirm whether IDO1^high^ ovarian cancer cell-derived EVs could promote angiogenesis through regulating endothelial mitophagy, we constructed a co-culture system of EVs-endothelial cells in vitro. Indeed, IDO1-overexpression ovarian cancer cell-derived EVs decreased the endothelial ATP content while IDO1-knockdown ovarian cancer cell-derived EVs increased the endothelial ATP content (Fig. 7a). Consistently, IDO1-overexpression ovarian cancer cell-derived EVs increased the endothelial mito-ROS, while IDO1-knockdown ovarian cancer cell-derived EVs suppressed the endothelial mito-ROS (Fig. 7b). A mitochondrial membrane potential assay with TMRE staining revealed that IDO1-overexpression ovarian cancer cell-derived EVs inhibited endothelial mitochondrial membrane potential (Fig. 7c). Taken together, these results suggested that IDO1^high^ ovarian cancer cell-derived EVs interrupted endothelial mitochondrial function, which might trigger mitophagy.

To further elucidate the role of IDO1^high^ ovarian cancer cell-derived EVs in endothelial mitophagy, endothelial cells were incubated with EVs obtained from IDO1-overexpression and -knockdown ovarian cancer cells. Western blot revealed that IDO1^high^ ovarian cancer cell-derived EVs upregulated the LC3II/LC3I ratio and IDO1 expression in endothelial cells (Fig. 7d). Moreover, mitochondria were isolated from endothelial cells in different intervention groups and Western blot revealed that IDO1^high^ ovarian cancer cell-derived EVs upregulated the expression of PINK1 and Parkin, which are classical markers of the mitophagy-associated pathway (Fig. 7e). Additionally, the expression of IDO1 was also found to be upregulated in these conditions (Fig. 7f). Therefore, these findings indicated that IDO1^high^ ovarian cancer cell-derived EVs activated PINK1-Parkin-dependent mitophagy.

Next, we verified whether IDO1^high^ ovarian cancer cell-derived EVs could regulate angiogenesis through activating endothelial mitophagy. Tube formation assays showed that the tube length of endothelial cells treated with IDO1^high^ ovarian cancer cell-derived EVs was markedly greater than that of the control group, and these effects could be rescued by HCQ (Fig. 7g). Besides, wound healing migration assay revealed that IDO1^high^ ovarian cancer cell-derived EVs promoted wound healing in endothelial cells compared with that in the control group, which could be reversed by HCQ (Fig. 7h). Therefore, the above findings indicated that IDO1^high^ ovarian cancer cell-derived EVs promoted angiogenesis through activating mitophagy of endothelial cells.

### IDO1^high^ cancer cell-derived EVs promoted endothelial mitophagy by increasing NAD + levels and sirt3 expression

**Fig. 8:**
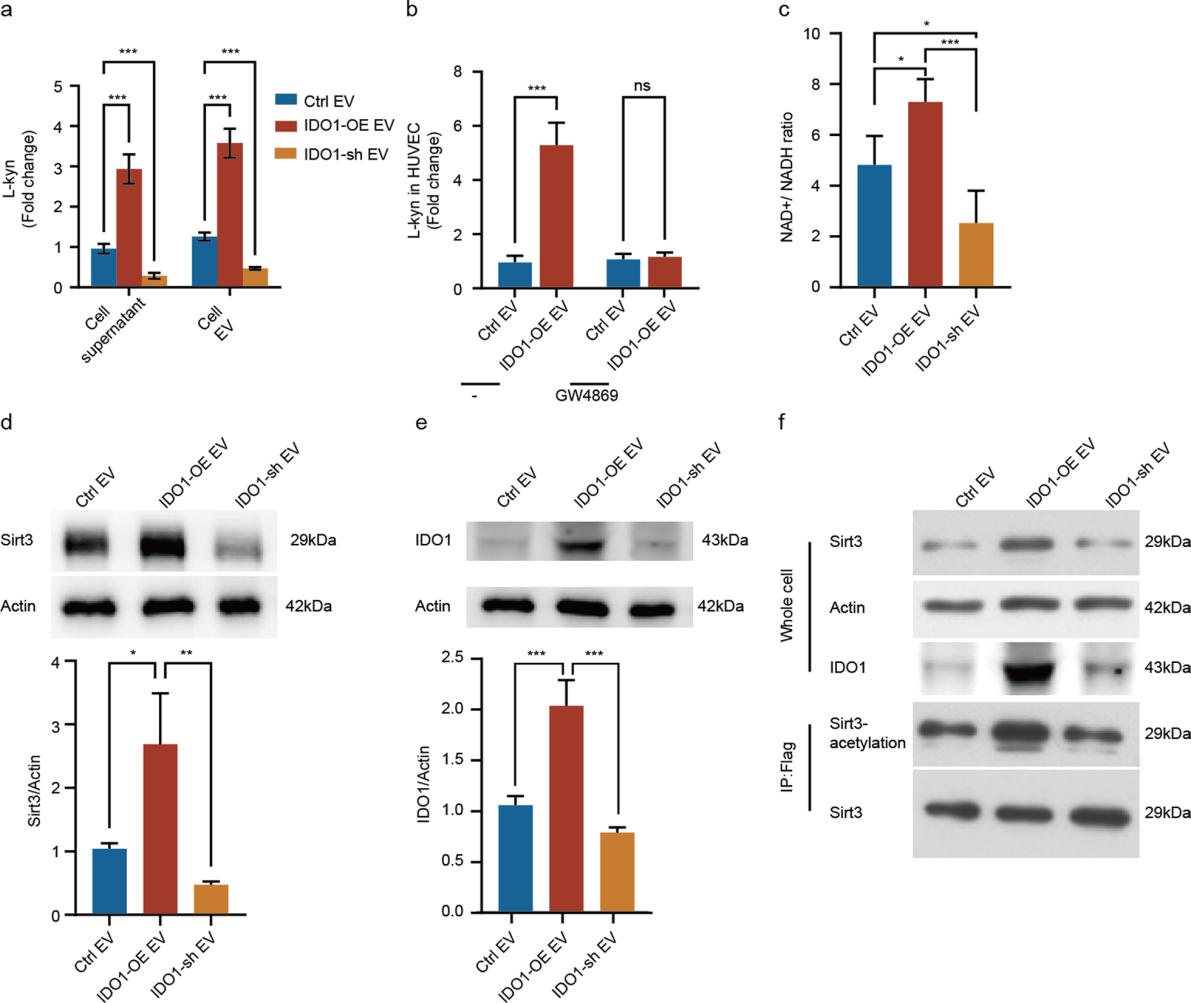
IDO1^high^ OC cell-derived EVs activated endothelial mitophagy by upregulating NAD + level and sirt3 expression. **a** Level of L-kyn in cell supernatant and cell derived-EVs of Ctrl EVs,IDO1-OE EVs or IDO1-sh EVs (n = 5, Mean ± SD, two-way ANOVA, ***P < 0.001). **b** Level of L-kyn in the endothelial cells incubated with different groups (n = 5, Mean ± SD, two-way ANOVA, ***P < 0.001, *ns.* no significance). **c** Level of NAD + /NADH in the endothelial cells treated with Ctrl EVs, IDO1-OE EVs or IDO1-sh EVs (n = 5, Mean ± SD, one-way ANOVA, *P < 0.05, ***P < 0.001). **d** Representative images and quantification analysis of sirt3 protein expression in the endothelial cells incubated with Ctrl EVs, IDO1-OE EVs or IDO1-sh EVs by Western blot (n = 3, Mean ± SD, one-way ANOVA, * *P* < 0.05,**P < 0.01). **e** Representative images and quantification analysis of IDO1 protein expression in the endothelial cells incubated with Ctrl EVs, IDO1-OE EVs or IDO1-sh EVs by Western blot (n = 3, Mean ± SD, one-way ANOVA, ***P < 0.001). **f** Co-immunoprecipitation (Co-IP) of sirt3 and sirt3-acetylation in endothelial cells after treatment with Ctrl EVs, IDO1-OE EVs or IDO1-sh EVs

To further clarify the molecular mechanisms by how IDO1^high^ ovarian cancer cell-derived EVs promote endothelial mitophagy, we first detected L-kyn levels in the culture supernatant and in EVs obtained from IDO1-overexpression and -knockdown ovarian cancer cells using ELISA assay. As expected, the results revealed that the L-kyn level was higher in the culture supernatant and EVs from IDO1- overexpression ovarian cancer cells than in those from IDO1-knockdown ovarian cancer cells or control ovarian cancer cells, indicating the aberrant upregulation of IDO1 in ovarian cancer cells contributed to the release of L-kyn-enriched EVs. Similarly, there existed a phenomenon of L-kyn enrichment in the EVs compared to the cell supernatant (Fig. 8a). It is well-accepted that EVs could regulate the biological functions of recipient cells by delivering their contents [36]. Thus, we assessed L-kyn level in the endothelial cells after cotreatment with EVs acquired from IDO1-overexpression ovarian cancer cells. Consistently, ELISA assay showed that EVs acquired from IDO1-overexpression ovarian cancer cells significantly increased the L-kyn level in the endothelial cells, and these effects were reversed by treatment with GW4869, a classical EV inhibitor (Fig. 8b). Collectively, this evidence indicated that IDO1 ^high^ ovarian cancer cells could deliver L-kyn to endothelial cells through EVs.

NAD +, a critical product involved in L-kyn metabolic pathway [56], was reported to activate mitophagy by regulating sirtuin family functions [57–59]. Thereby, we inferred that the increase in the intracellular L-kyn concentration in endothelial cells induced by EVs released from IDO1^high^ ovarian cancer cells might lead to an increase of NAD + , which could participate in modulating mitophagy. In order to verify the hypothesis, the NAD + level in the treated endothelial cells were detected, and the results displayed that IDO1-overexpression ovarian cancer cell-derived EVs increased NAD + /NADH ratio in endothelial cells compared with that in endothelial cells incubated with IDO1-knockdown ovarian cancer cell-derived EVs or IDO1-control ovarian cancer cell-derived EVs (Fig. 8c).

Sirtuin3 (sirt3), an NAD + -dependent deacetylase, is essential for regulating mitochondrial acetylation levels associated with mitophagy [58–60]. In addition to increasing NAD + level, we supposed that IDO1^high^ ovarian cancer cell-derived EVs might drive abnormal Sirt3 expression linked to mitophagy in endothelial cells. Unsurprisingly, Western blot revealed that IDO1-overexpression ovarian cancer cell-derived EVs upregulated endothelial sirt3 expression compared to that in IDO1-knockdown or -control ovarian cancer cell-derived EVs (Fig. 8d). Additionally, these IDO1-overexpression ovarian cancer cell-derived EVs were also found to enhance endothelial IDO1expression, in contrast to EVs from IDO1-knockdown or -control ovarian cancer cells (Fig. 8e). Acetylation could affect protein stability [61], and we further performed a co-immunoprecipitation assay to test whether IDO1^high^ ovarian cancer cell-derived EVs could upregulate sir3 expression by increasing the acetylation levels. Consistently, the results uncovered that increased sirt3 and IDO1 expression and enhanced sirt3 acetylation levels in the endothelial cells treated with IDO1-overexpression ovarian cancer cell-derived EVs compared with treated with IDO1-knockdown or -control ovarian cancer cell-derived EVs (Fig. 8f). In summary, the above findings suggested that IDO1^high^ ovarian cancer cell-derived EVs increased endothelial NAD + levels involved in regulating sirt3 functions as well as upregulated endothelial sirt3 expression through enhancing the acetylation levels, which closely correlated with mitophagy process.

### IDO1 inhibitor suppressed endothelial mitophagy and effectively inhibited ovarian cancer progression

**Fig. 9:**
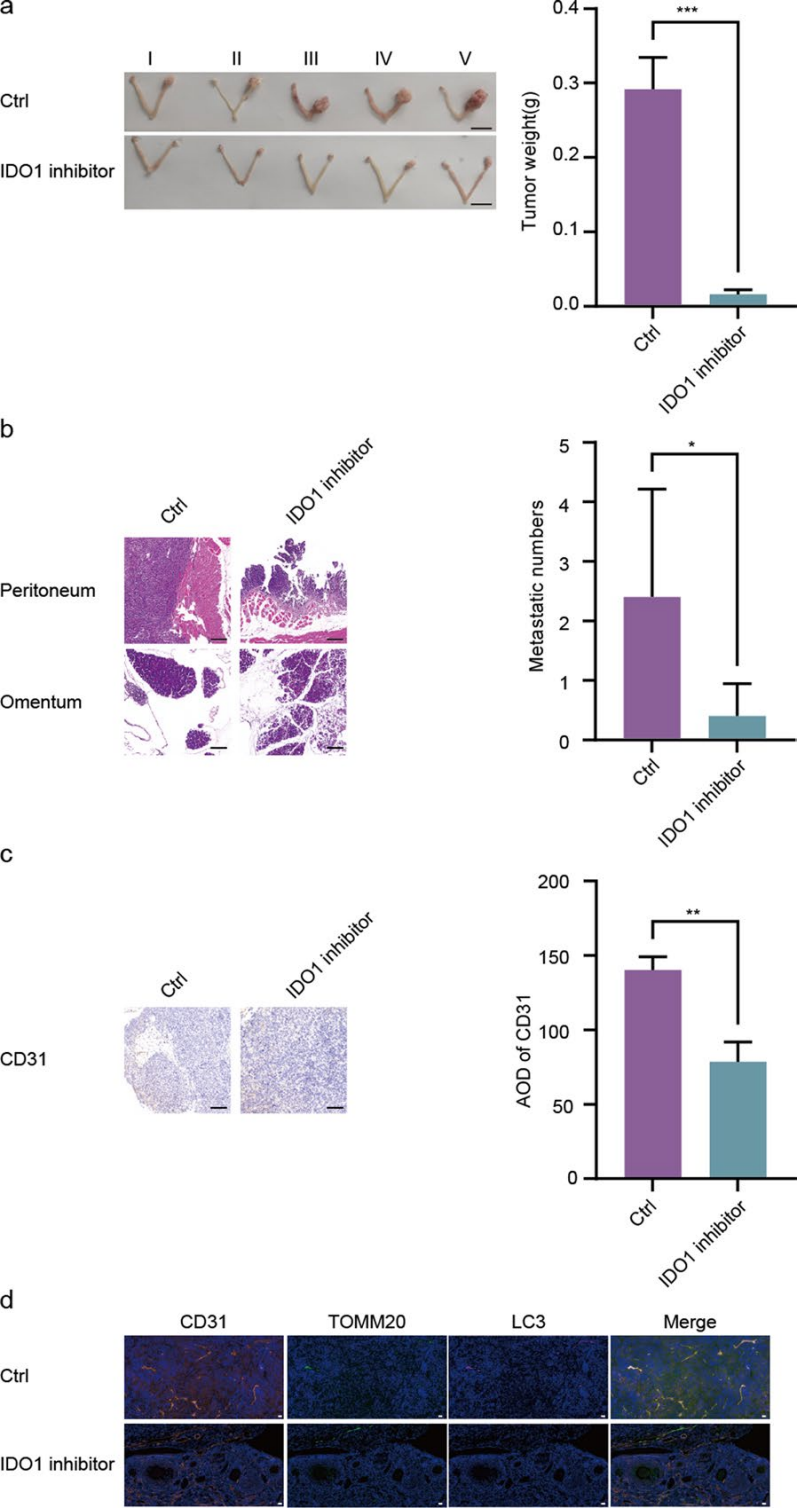
IDO1 inhibitor suppressed endothelial mitophagy and effectively inhibited ovarian cancer progression. **a** Representative images of the primary ovary tumor size in orthotopic OC model education with the ctrl (control) or IDO1 inhibitor. bar = 1 cm. (n = 5, Mean ± SD, t-test, ***P < 0.001). **b** Numbers of peritoneal metastases confirmed by HE staining in orthotopic OC model treated with ctrl or IDO1 inhibitor. Scale bar:50 μm. (n = 5, Mean ± SD, t-test, *P < 0.05). **c** Immunohistochemistry (IHC) analysis of CD31 in primary tumor tissues of orthotopic OC model education with the ctrl or IDO1 inhibitor. Scale bar:50 μm. (n = 3, Mean ± SD, t-test, **P < 0.01). **d** Representative images of LC3, TOMM20, and CD31 in primary tumor tissue sections of orthotopic OC model treated with the ctrl or IDO1 inhibitor by immunofluorescence staining. Scale bar:20 μm

Next, we developed a mouse orthotopic model of ovarian cancer to investigate the potential therapeutic value of IDO1.Two weeks after OC orthotopic implantation, the mice were treated with or without 1- Methyl-DL-tryptophan for 6 weeks. After that, the mice were euthanized and tumor tissues were excised for further analysis. The results revealed that, compared with the control group, the IDO1 inhibitor suppressed tumor formation, metastasis and angiogenesis (Fig. 9a–c). Consistently, multicolor immunohistochemistry showed that the IDO1 inhibitor decreased the colocalization of CD31 with LC3 and TOMM20 in primary tumor tissues of the orthotopic OC mouse model (Fig. 9d). Therefore, our data suggested that the IDO1 inhibitor repressed endothelial mitophagy involved in tumor angiogenesis and development in the orthotopic OC mice model.

## Discussion

**Fig. 10:**
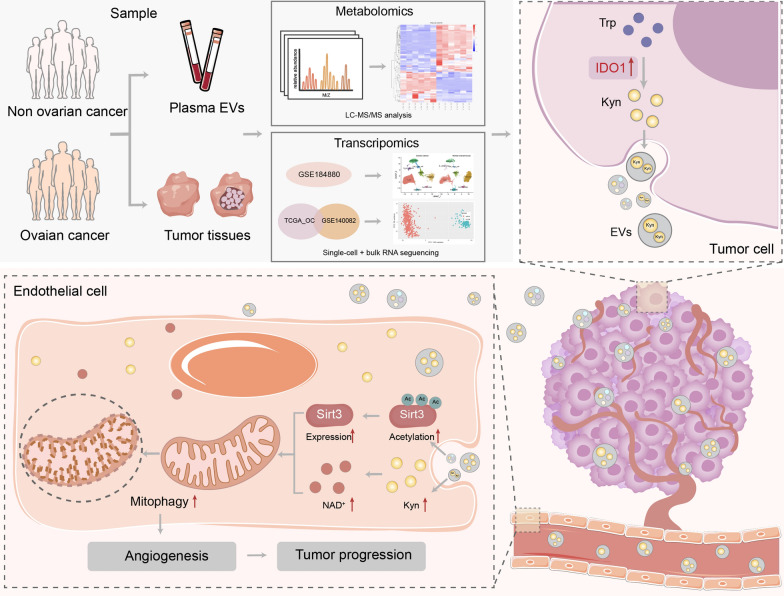
Graphic abstract

Although angiogenesis, which can be regulated by EVs, is generally considered an essential hallmark of tumor progression, the exact mechanism is still not entirely clarified. Mitophagy, a type of selective autophagy, has been identified to be associated with endothelial function regulation [23, 26, 62], while its biological role in tumor angiogenesis is less elucidated. Here, we explored the role of tumor EVs in regulating endothelial mitophagy related to OC angiogenesis and progression. Through a series of in vivo and in vitro experiments, we revealed that ovarian cancer cell-derived EVs facilitated angiogenesis by activating endothelial mitophagy. Through integrating multi-omics analyses, we speculated that abnormal IDO1 upregulation of tumor cells involved in anomalous tryptophan metabolism could release L-kyn enriched EVs, which may partly lead to a high level of L-kyn in EVs derived from tumor tissues and plasma from OC patients, as validated in another clinical cohort. Mechanistically, IDO1 ^high^ ovarian cancer cell-derived EVs regulated NAD + level and sirt3 acetylation of endothelial cells, which correlated with the mitophagy modulation (Fig. 10). Notably, the IDO1 inhibitor could suppress endothelial mitophagy and tumor progression in the OC orthotopic mouse model.

In the current study, we found that mitophagy of endothelial cells could be activated by tumor cell-derived EVs, playing important roles in OC angiogenesis and progression. Previous studies have indicated that tumor cell-derived EVs could serve as a vital mediator for intercellular crosstalk in regulating angiogenesis and disease progression [9, 63]. Additionally, mitophagy has been demonstrated as an important biological mechanism mediating vascular structure and function by regulating endothelial cells [23–26]. We found that cancer cell-derived EVs could facilitate endothelial mitophagy, tumor angiogenesis, and development in an orthotopic OC mice model. Further, cancer cell-derived EVs could promote the tube formation ability of endothelial cells by activating mitophagy. Therefore, our study is the first to indicate that mitophagy acted as an essential regulator in tumor angiogenesis and progression, which could be modulated by ovarian cancer cell-derived EVs.

Further, we identified that aberrantly high expression levels of IDO1 related to tryptophan metabolic abnormalities in tumor cells could release L-kyn enriched EVs, contributing to increased NAD + levels of endothelial cells, which might be involved in mitophagy activation. EV contents are generally demonstrated to be similar to those of parental cells [36]. It has also been widely indicated that IDO1, the first rate-limiting enzyme in the tryptophan-kynurenine (Trp-kyn) metabolism pathway, is upregulated in various cancer tissues, including ovarian cancer, and is related to poor patient prognosis [64–67]. Our findings are consistent with the previous studies and suggested that IDO1 over-expressed in tumor cells associated with Trp-kyn metabolic reprogramming could increase secretion of L-kyn enriched EVs. Moreover, numerous studies have demonstrated that EVs mediate intercellular communication by transferring their contents [36]. Our findings also suggested that EVs released from tumor cells could deliver their contents to endothelial cells and exert biological effects. Interestingly, unlike the major previous studies that mainly focus on the RNA and protein cargos in EVs, we observed that EVs could mediate intercellular communication via enriching as well as transmitting metabolites such as L-kyn. Kyn is an essential substrate for the de novo NAD^+^ synthesis pathway. In addition, NAD^+^ is known as a cofactor contributing to mitochondrial functions including mitophagy [55]. Here, we demonstrated that EVs obtained from IDO1 ^high^ tumor cells increased L-kyn levels of the endothelial cells, which could further promote the intercellular NAD + levels, implying part of the mechanisms contributing to mitophagy. These findings suggest that metabolic contents in EVs should be taken into account as an important regulator of intercellular communication.

In addition, abnormally high expression levels of IDO1 in tumor cells involved in tryptophan metabolic disorder could secrete L-kyn enriched EVs into the TME and circulation, resulting in elevated L-kyn enriched EVs in tumor tissues and plasma with the patients, which might furnish evidence for OC diagnosis. Previous studies have suggested that EVs incorporate abundant metabolites [40, 68–72], representing an undeveloped fountainhead for biomarker identification. In addition, altered metabolism patterns of tumor cells could influence EV metabolome. Consistently, our findings indicated that an upregulation of L-kyn in EVs released from IDO1 high tumor cells associated with tryptophan metabolic reprogramming could further result in high levels of L-kyn in EVs derived from tumor tissues and plasma with OC patients. We therefore, speculated that L-kyn could constitute a potential EV-based metabolomic fingerprint, offering evidence for the development of non-invasive biomarkers in OC.

Ultimately, we uncovered that sirt3 expression of the endothelial cells could be upregulated by EVs released from IDO1^high^ ovarian cancer cells, which is known to play essential roles in regulating mitophagy [60, 73, 74]. Prior studies have shown that acetylation favors protein stabilization [75]. Accordingly, we further indicated that IDO1^high^ ovarian cancer cell derived-EVs could upregulate sirt3 expression of the endothelial cells by increasing the acetylation modification levels. Moreover, the IDO1 inhibitor could suppress endothelial mitophagy, tumor angiogenesis and progression in orthotopic OC mice.

Inevitably, several limitations might affect the findings obtained. To begin with, we were unable to identify the potential diagnostic value of EV L-kyn in ovarian cancer due to a lack of sufficient clinical samples. In addition, we did not clarify the underlying roles of sirt3 modulated by IDO1^high^ ovarian cancer cell-derived EVs in regulating endothelial mitophagy, which will be an essential issue for future research. Studies indicated that IDO1 inhibitors modulate the tumor immune microenvironment, encompassing ovarian cancer [76, 77]. Thus, the effects of IDO1 inhibitor-mediated modulation of endothelial mitophagy on the immune cells within the tumor microenvironment need to be further explored.

## Conclusions

In summary, we revealed for the first time that cancer cell-derived EVs activated endothelial mitophagy, which was related to tumor angiogenesis and progression in ovarian cancer, providing novel insights into the role of endothelial mitophagy in tumor angiogenesis. Further, the abnormally high IDO1 expression in cancer cells, associated with tryptophan metabolic reprogramming, could contribute to L-kyn changes in EVs, resulting in increased L-kyn levels in EVs obtained from plasma and the TME in OC patients. Mechanistically, IDO1^high^ cancer cell-derived EVs promoted mitophagy by delivering L-kyn or increasing sirt3 acetylation levels of the endothelial cells. Thus, metabolites in EVs represent not only a promising biomarker but also a key to elucidating the molecular mechanisms of tumor development in OC.

### Supplementary Information

**Additional file 1: Figure S1.** Characterization and internalization of EVs. **a** Images of CD9, CD81 and CD63 protein expression in cell-derived EVs by Western blot. **b** Images of labeled EVs endocytosed by endothelial cells. Scale bar:10 μm. **Figure S2.** OC cell-derived EVs promoted angiogenesis by activating mitophagy of endothelial cells. **a** Representative images and quantitation of mitotracker, LC3 and DAPI in endothelial cells treated with different groups imaged by confocal microscopy. Scale bar:10 μm. (n = 5, Mean ± SD, one-way ANOVA, ***P ＜ 0.001). **b,** Representative images and quantitative analysis of tube formation in different groups(n=5). Scale bar: 500 μm. (n = 5, Mean ± SD, one-way ANOVA, ***P ＜ 0.001). **Figure S3.** Metabolic profiles characterization of EVs from OC and non-OC plasma. **a** Images of CD9 and CD81 expression in plasma-derived EVs from OC and non-OC patients by Western blot. **b** QC sample correlation analysis of positive and negative ion mode. **c** Principal component analysis (PCA) of metabolome samples in negative ion mode. **d** Partial Least Squares Discrimination Analysis (PLS-DA) of metabolome samples in positive and negative ion mode. **e** Pie chart depicting classification of metabolites in negative ion mode. **f** Clustering heatmap of differential metabolites in positive and negative ion mode. **g** KEGG pathway enrichment of differentially accumulated metabolites in negative ion mode between OC and non-OC plasma derived-EVs. For **b**–**g** all n = 6 plasma-derived EVs from patients with OC or non-OC were performed for analysis. **Figure S4.** Clustering of cellular landscape between OC and non-OC samples by analyzing scRNA-seq data. **a** T-Distributed Stochastic Neighbor Embedding（tSNE）revealing 8 clusters by integrating analysis of OC and non-OC samples. **b** Percentage of major cell clusters in OC samples versus non-OC ovarian samples. **c** Percentage of epithelial cell sub-clusters in OC samples versus non-OC ovarian samples. **d** Distribution of* IDO1 *in epithelial cell sub-clusters of OC samples versus non-OC ovarian samples. For **a**–**d** all n = 7 OC and n = 5 non-OC samples obtained from GSE184880 dataset were used for analysis. **Figure S5.** Identification of hub genes associated with tryptophan metabolism in OC. **a** Heatmap showing differentiate expression genes (DEGs) in OC samples from TCGA ovarian cancer dataset and non-OC samples from GETx dataset. **b** Univariate Cox regression analysis indicating tryptophan metabolism related genes involving in OC prognosis of OCGA_OV dataset. **Figure S6.** Construction and validation of IDO1 over-expression and down-expression in the OC cell line. **a** Validation for IDO1 over-expression and down-expression in A2780 by RT-PCR (n = 3, Mean ± SD, one-way ANOVA, **P ＜ 0.01, ***P ＜ 0.001). **b** Validation for IDO1 over-expression and down-expression in A2780 by Western blot(n = 3, Mean ± SD, one-way ANOVA, *P ＜ 0.05, *P ＜ 0.01,***P ＜ 0.001). **Table S1.** Primary antibodies for Western blot, IHC or CoIP. **Table S2.** Primer sequence.

## Data Availability

Any data generated or analyzed during this article can be available from the corresponding author upon reasonable request.

## Abbreviations

ATP: Adenosine 5′-triphosphate
BSA: Bovine serum albumin
CoIP: Co-immunoprecipitation
DEGs: Differentially expressed genes
ELISA: Enzyme linked immunosorbent assay
EVs: Extracellular vesicles
FDR: False discovery rate
GEO: Gene expression omnibus
GSVA: Gene set variation analysis
HCQ: Hydroxychloroquine
HE: Hematoxylin–eosin
HPA: Human protein atlas database
HUVECs: Human umbilical vein endothelial cells
IHC: Immunohistochemistry
IDO1: Indoleamine 2,3 dioxygenase-1
LC3: Microtubule associated protein 1 light chain 3 alpha
KEGG: Kyoto Encyclopedia of Genes and Genomes
L-kyn: l-kynurenine
Mito-ROS: Mitochondrial reactive oxygen species
MSigDB: Molecular Signature Database
NAD +: Nicotinamide adenine dinucleotide
NADH: Nicotinamide adenine dinucleotide
OC: Ovarian cancer
Parkin: Parkin RBR E3 ubiquitin protein ligase
PBS: Phosphate-buffered saline
PCA: Principal component analysis
PINK1: PTEN induced kinase 1
PLS-DA: Partial least squares discrimination analysis
PVDF: Polyvinylidene difluoride
QC: Quality control
RT: Room temperature
ScRNA-seq: Single-cell RNA-seq
Sirt3: Sirtuin3
STR: Short tandem repeat
Trp-kyn: Tryptophan-kynurenine
TEM: Transmission electron microscopy
TMAs: Tissue microarrays
TME: Tumor microenvironment
TMRE: Tetramethyl rhodamine ethyl ester
TOMM20: Translocase of outer mitochondrial membrane 20
T-SNE: T-distributed stochastic neighbor embedding
UMAP: Uniform manifold approximation and projection
