# Supplementary material for: Kynurenine in IDO1high cancer cell-derived extracellular vesicles promotes angiogenesis by inducing endothelial mitophagy in ovarian cancer
Source: J Transl Med. 2024 Mar 11;22:267. doi: 10.1186/s12967-024-05054-5 (PMC10929174; doi:10.1186/s12967-024-05054-5)
Supplement: Supplementary file 1 — Additional file 1: Figure S1. Characterization and internalization of EVs. a Images of CD9, CD81 and CD63 protein expression in cell-derived EVs by Western blot. b Images of labeled EVs endocytosed by endothelial cells. Scale bar:10 μm. Figure S2. OC cell-derived EVs promoted angiogenesis by activating mitophagy of endothelial cells. a Representative images and quantitation of mitotracker, LC3 and DAPI in endothelial cells treated with different groups imaged by confocal microscopy. Scale bar:10 μm. (n = 5, Mean ± SD, one-way ANOVA, ***P ＜ 0.001). b, Representative images and quantitative analysis of tube formation in different groups(n=5). Scale bar: 500 μm. (n = 5, Mean ± SD, one-way ANOVA, ***P ＜ 0.001). Figure S3. Metabolic profiles characterization of EVs from OC and non-OC plasma. a Images of CD9 and CD81 expression in plasma-derived EVs from OC and non-OC patients by Western blot. b QC sample correlation analysis of positive and negative ion mode. c Principal component analysis (PCA) of metabolome samples in negative ion mode. d Partial Least Squares Discrimination Analysis (PLS-DA) of metabolome samples in positive and negative ion mode. e Pie chart depicting classification of metabolites in negative ion mode. f Clustering heatmap of differential metabolites in positive and negative ion mode. g KEGG pathway enrichment of differentially accumulated metabolites in negative ion mode between OC and non-OC plasma derived-EVs. For b–g all n = 6 plasma-derived EVs from patients with OC or non-OC were performed for analysis. Figure S4. Clustering of cellular landscape between OC and non-OC samples by analyzing scRNA-seq data. a T-Distributed Stochastic Neighbor Embedding（tSNE）revealing 8 clusters by integrating analysis of OC and non-OC samples. b Percentage of major cell clusters in OC samples versus non-OC ovarian samples. c Percentage of epithelial cell sub-clusters in OC samples versus non-OC ovarian samples. d Distribution of IDO1 in epithelial cell sub-cluster [file 12967_2024_5054_MOESM1_ESM.docx]

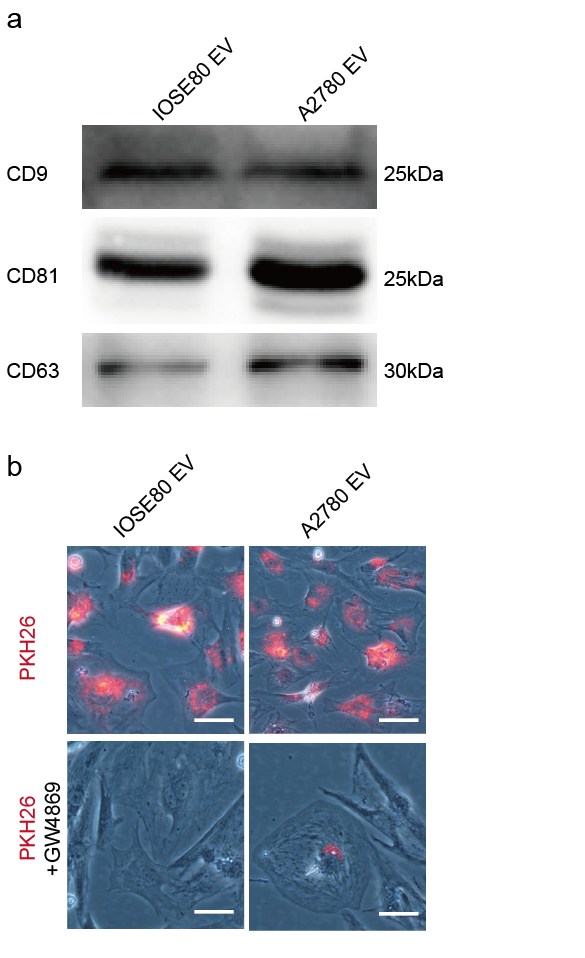


### Fig. S1 Characterization and internalization of EVs

**a,** Images of CD9, CD81 and CD63 protein expression in cell-derived EVs by Western blot. **b,** Images of labeled EVs endocytosed by endothelial cells. Scale bar:10μm.


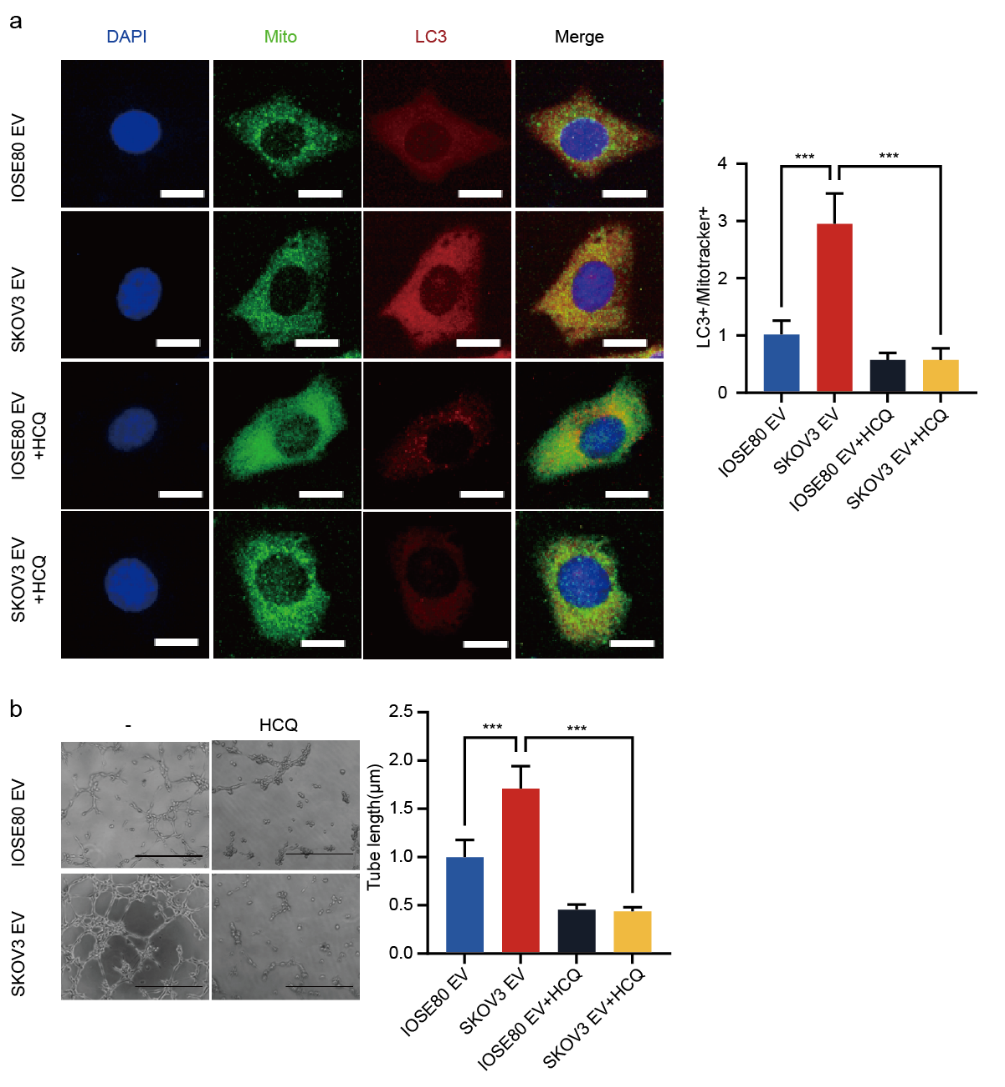


### Fig. S2 OC cell-derived EVs promoted angiogenesis by activating mitophagy of endothelial cells.

**a,** Representative images and quantitation of mitotracker, LC3 and DAPI in endothelial cells treated with different groups imaged by confocal microscopy. Scale bar:10μm. (n=5, Mean±SD, one-way ANOVA, ****P*＜0.001). **b,** Representative images and quantitative analysis of tube formation in different groups(n=5). Scale bar: 500μm. (n=5, Mean±SD, one-way ANOVA, ****P*＜0.001)


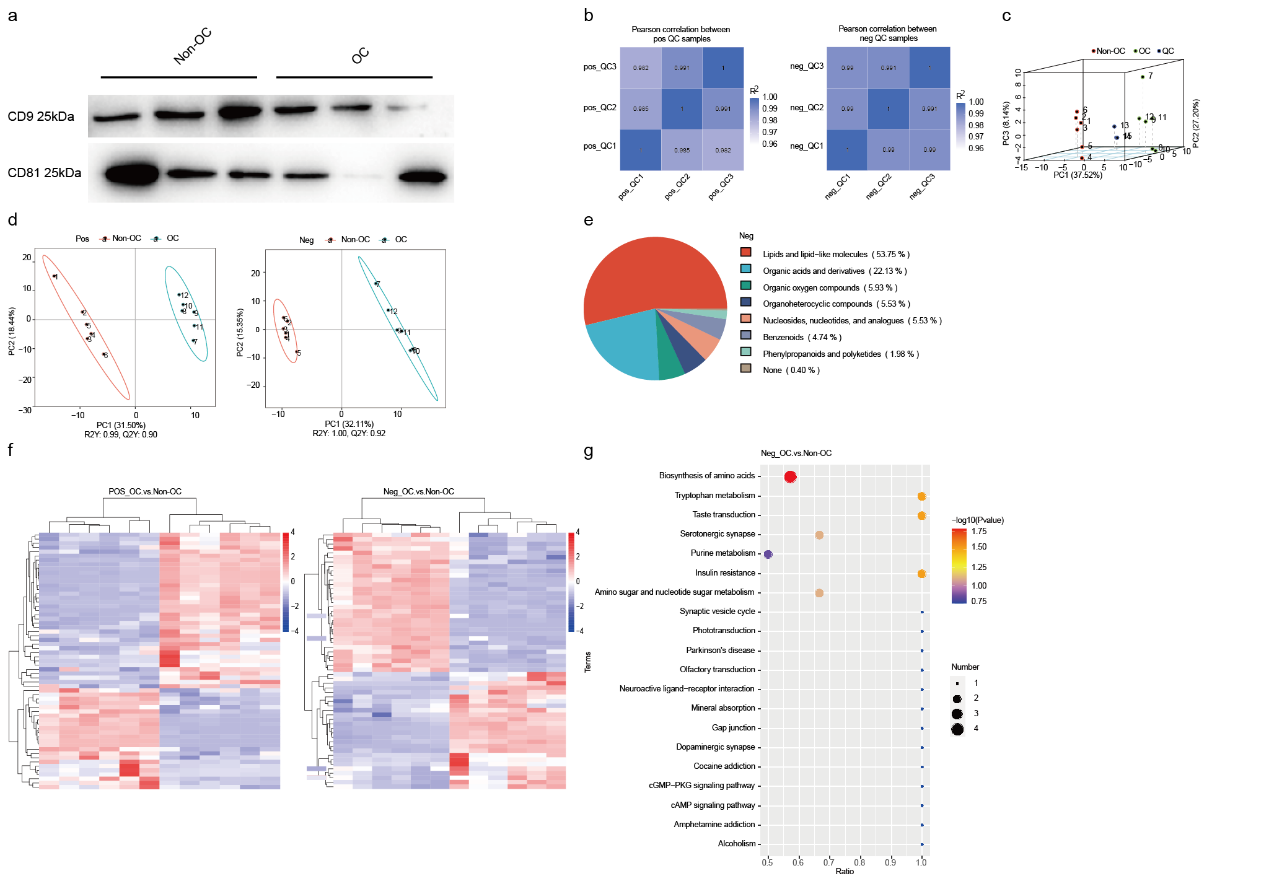


### Fig. S3 Metabolic profiles characterization of EVs from OC and non-OC plasma.

**a,** Images of CD9 and CD81 expression in plasma-derived EVs from OC and non-OC patients by Western blot. **b,** QC sample correlation analysis of positive and negative ion mode. **c,** Principal component analysis (PCA) of metabolome samples in negative ion mode. **d,** Partial Least Squares Discrimination Analysis (PLS-DA) of metabolome samples in positive and negative ion mode. **e,** Pie chart depicting classification of metabolites in negative ion mode. **f,** Clustering heatmap of differential metabolites in positive and negative ion mode. **g,** KEGG pathway enrichment of differentially accumulated metabolites in negative ion mode between OC and non-OC plasma derived-EVs. **For b-g,** all n=6 plasma-derived EVs from patients with OC or non-OC were performed for analysis.


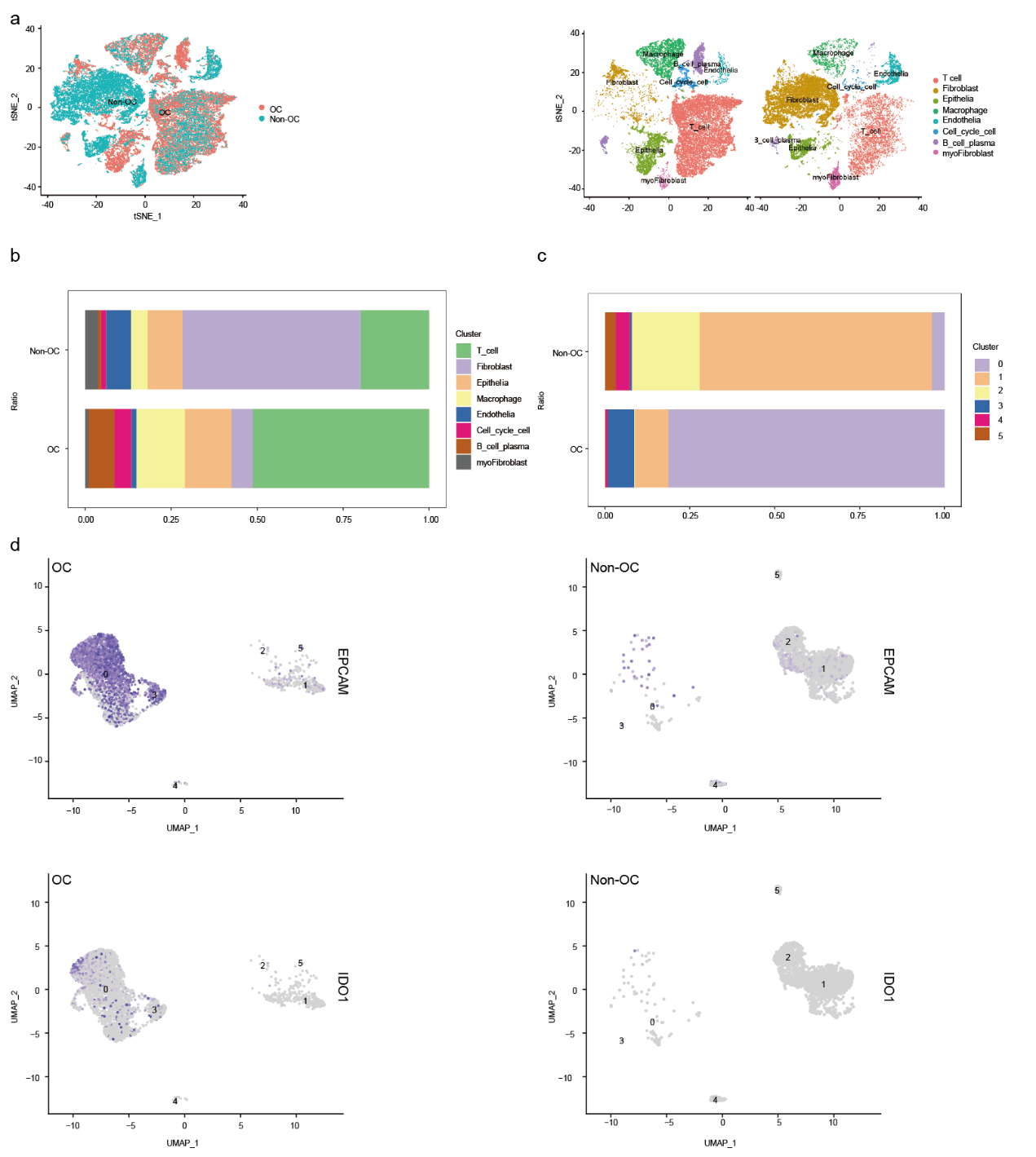


### Fig. S4 Clustering of cellular landscape between OC and non-OC samples by analyzing scRNA-seq data

**a,** T-Distributed Stochastic Neighbor Embedding（tSNE）revealing 8 clusters by integrating analysis of OC and non-OC samples. **b,** Percentage of major cell clusters in OC samples versus non-OC ovarian samples. **c,** Percentage of epithelial cell sub-clusters in OC samples versus non-OC ovarian samples. **d,** Distribution of*IDO1* in epithelial cell sub-clusters of OC samples versus non-OC ovarian samples. **For a-d,** all n=7 OC and n=5 non-OC samples obtained from GSE184880 dataset were used for analysis.


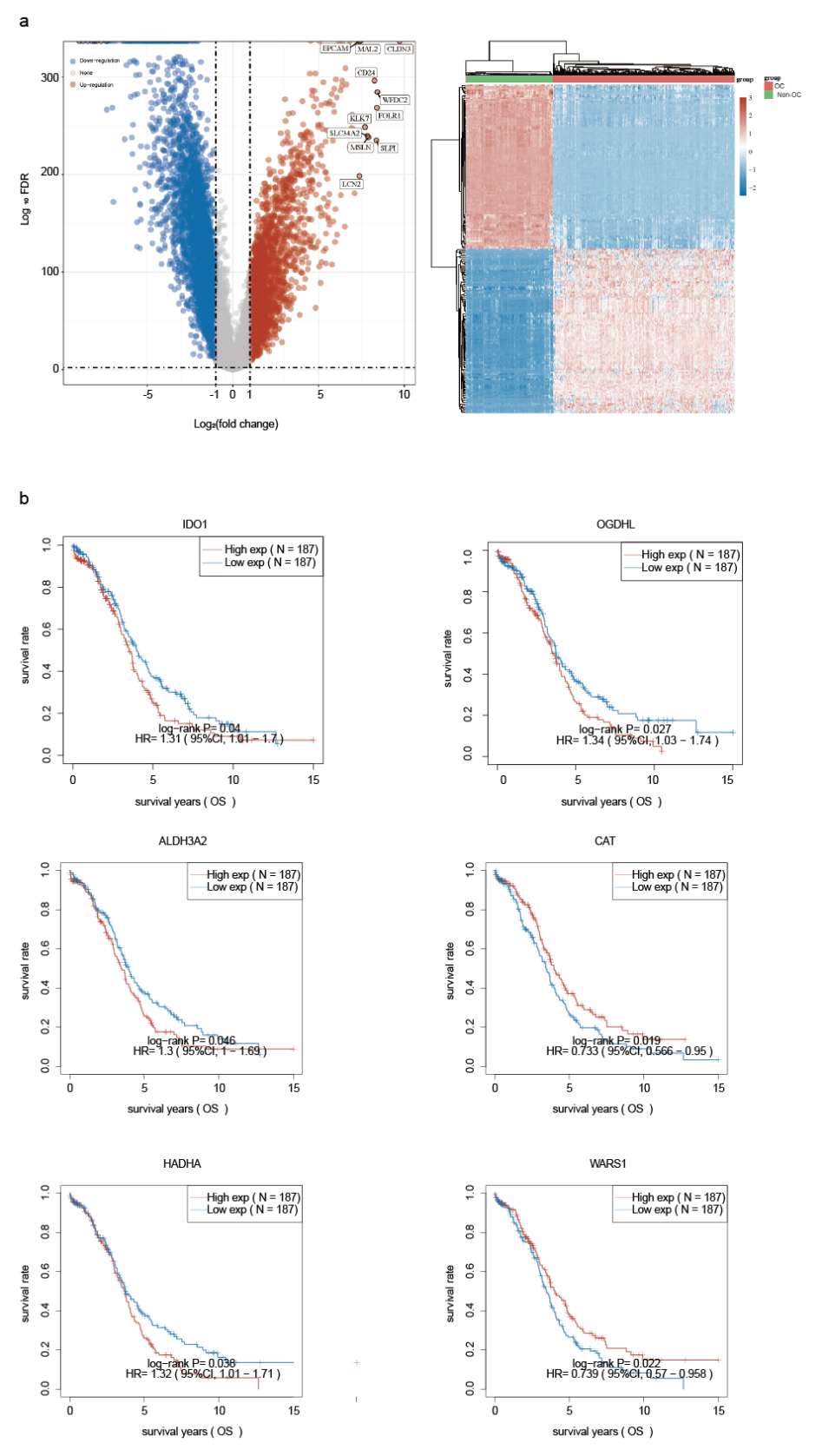


### Fig. S5 Identification of hub genes associated with tryptophan metabolism in OC

**a,** Heatmap showing differentiate expression genes (DEGs) in OC samples from TCGA ovarian cancer dataset and non-OC samples from GETx dataset. **b,** Univariate Cox regression analysis indicating tryptophan metabolism related genes involving in OC prognosis of OCGA_OV dataset.


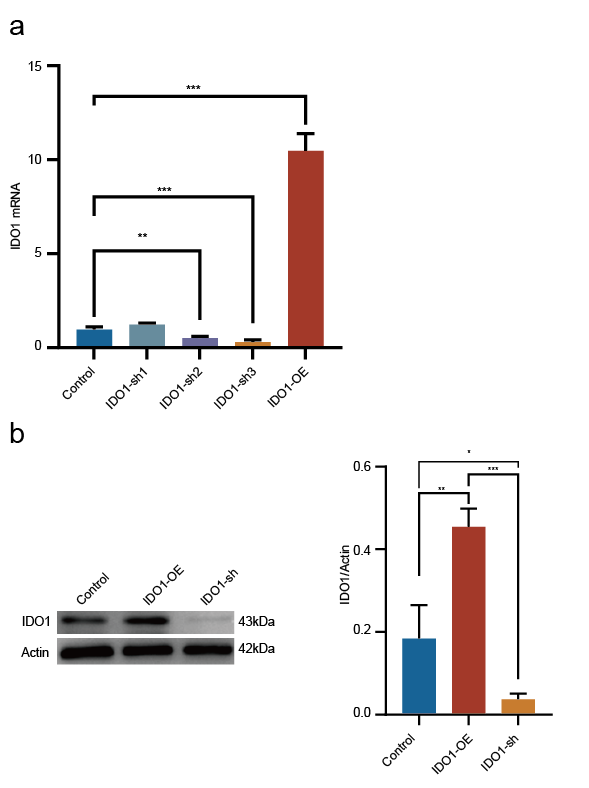


### Fig. S6 Construction and validation of IDO1 over-expression and down-expression in the OC cell line.

**a,** Validation for IDO1 over-expression and down-expression in A2780 by RT-PCR (n=3, Mean±SD, one-way ANOVA, ***P*＜0.01, ****P*＜0.001). **b,** Validation for IDO1 over-expression and down-expression in A2780 by Western blot(n=3, Mean±SD, one-way ANOVA, **P*＜0.05, **P*＜0.01,****P*＜0.001).

### Table S1. Primary antibodies for Western blot, IHC or CoIP

| **Primary antibodies** | **SOURCE** | **IDENTIFIER** | **Application** |  |
| --- | --- | --- | --- | --- |
| CD9 | Abcam | Cat# ab236630 | WB |  |
| CD31 | Abcam | Cat# ab281583 | IHC |  |
| CD63 | Abcam | Cat# ab134045 | WB |  |
| CD81 | Abcam | Cat #ab109201 | WB |  |
| LC3 | Abcam | Cat #ab192890 | WB |  |
| PINK1 | Abcam | Cat# ab216144 | WB |  |
| Parkin | Abcam | Cat #ab77924 | WB |  |
| Sirt3 | Abcam | Cat# ab246522 | WB/IP |  |
| IDO1 | Abcam | Cat# ab211017 | WB |  |
| IDO1 | Proteintech | Cat# 13268-1-AP | IHC |  |
| Actin | Proteintech | Cat # 81115-1-RR | WB |  |
| Tublin | Proteintech | Cat# 11224-1-AP | WB |  |
| TOMM20 | Abcam | Cat# ab186735 | WB |  |
| Acetylated-Lysine Mab | abmart | Cat# M30069M | IP |  |

### Table S2. Primer sequence

| **Gene** | **Primer sequence** | **Application** |
| --- | --- | --- |
| IDO1 | Forward Primer: GCCTGATCTCATAGAGTCTGGC | qRT-PCR |
|  | Reverse Primer: TGCATCCCAGAACTAGACGTGC |  |
| GAPDH | Forward Primer: GTCTCCTCTGACTTCAACAGCG | qRT-PCR |
|  | Reverse Primer:ACCACCCTGTTGCTGTAGCCAA |  |
